# Long-term persistence of supernumerary B chromosomes in multiple species of *Astyanax* fish

**DOI:** 10.1186/s12915-021-00991-9

**Published:** 2021-03-19

**Authors:** Duílio Mazzoni Zerbinato de Andrade Silva, Francisco J. Ruiz-Ruano, Ricardo Utsunomia, María Martín-Peciña, Jonathan Pena Castro, Paula Paccielli Freire, Robson Francisco Carvalho, Diogo T. Hashimoto, Alexander Suh, Claudio Oliveira, Fábio Porto-Foresti, Roberto Ferreira Artoni, Fausto Foresti, Juan Pedro M. Camacho

**Affiliations:** 1 Departamento de Biologia Estrutural e Funcional, Instituto de Biociências de Botucatu, Universidade Estadual Paulista, UNESP, Distrito de Rubião Junior, Botucatu, SP 18618-970 Brazil; 2 Department of Organismal Biology – Systematic Biology, Evolutionary Biology Centre, Uppsala University, SE-752 36 Uppsala, Sweden; 3 Departamento de Genética, Universidad de Granada, 18071 Granada, Spain; 4 School of Biological Sciences, University of East Anglia, Norwich Research Park, Norwich, NR4 7TU UK; 5 Departamento de Genética, Instituto de Ciências Biológicas e da Saúde, ICBS, Universidade Federal Rural do Rio de Janeiro, Seropédica, RJ 23897-000 Brazil; 6 Departamento de Ciências Biológicas, Faculdade de Ciências, Universidade Estadual Paulista, UNESP, Campus de Bauru, Bauru, SP 17033-360 Brazil; 7 Departamento de Genética e Evolução, Universidade Federal de São Carlos, UFSCAR, São Carlos, SP 13565-905 Brazil; 8 Departamento de Biologia Estrutural, Molecular e Genética, Universidade Estadual de Ponta Grossa, UEPG, Ponta Grossa, PR 84030-900 Brazil; 9 Departamento de Imunologia, Instituto de Ciências Biomédicas, Universidade de São Paulo, USP, São Paulo, SP 05508-900 Brazil; 10 Centro de Aquicultura, Universidade Estadual Paulista, UNESP, Campus Jaboticabal, Jaboticabal, SP 14884-900 Brazil

**Keywords:** B chromosomes, *Astyanax*, Evolution, Genomics, Transcriptomics

## Abstract

**Background:**

Eukaryote genomes frequently harbor supernumerary B chromosomes in addition to the “standard” A chromosome set. B chromosomes are thought to arise as byproducts of genome rearrangements and have mostly been considered intraspecific oddities. However, their evolutionary transcendence beyond species level has remained untested.

**Results:**

Here we reveal that the large metacentric B chromosomes reported in several fish species of the genus *Astyanax* arose in a common ancestor at least 4 million years ago. We generated transcriptomes of *A. scabripinnis* and *A. paranae* 0B and 1B individuals and used these assemblies as a reference for mapping all gDNA and RNA libraries to quantify coverage differences between B-lacking and B-carrying genomes. We show that the B chromosomes of *A. scabripinnis* and *A. paranae* share 19 protein-coding genes, of which 14 and 11 were also present in the B chromosomes of *A. bockmanni* and *A. fasciatus*, respectively. Our search for B-specific single-nucleotide polymorphisms (SNPs) identified the presence of B-derived transcripts in B-carrying ovaries, 80% of which belonged to *nobox*, a gene involved in oogenesis regulation. Importantly, the B chromosome *nobox* paralog is expressed > 30× more than the A chromosome paralog. This indicates that the normal regulation of this gene is altered in B-carrying females, which could potentially facilitate B inheritance at higher rates than Mendelian law prediction.

**Conclusions:**

Taken together, our results demonstrate the long-term survival of B chromosomes despite their lack of regular pairing and segregation during meiosis and that they can endure episodes of population divergence leading to species formation.

**Supplementary Information:**

The online version contains supplementary material available at 10.1186/s12915-021-00991-9.

## Background

Thousands of eukaryotic species harbor dispensable B chromosomes which perpetuate in natural populations as genomic parasites [1, 2]. Recent research has shown that B chromosomes usually carry redundant genetic information already present on the standard (A) chromosomes [3–6]. The most remarkable exception is the paternal sex ratio (PSR) B chromosome in the wasp *Nasonia vitripennis*, which carries one gene (named *haploidizer*), which does not match to any sequence in standard (A) chromosomes [7]. In some cases, B chromosomes might have become useful elements for the host genome, as reported in the fungus *Nectria haematococca* where B chromosomes carry genes determining pathogenicity to pea [8]. In other cases, the possible evolution of Y chromosomes from pre-existing B chromosomes was suggested in the homopteran *Rhinocola aceris* and *Cacopsylla peregrina* [9, 10], and a similar derivation has been claimed for the germline-restricted chromosomes in songbirds [11]. B chromosomes are usually polymorphic within populations, but apparently similar Bs have been found in several closely related species, suggesting that they persisted across speciation events [12].

The genus *Astyanax* Baird & Girard is widely distributed from southern USA to central Argentina [13]. It includes about 200 species thus being one of the most diverse genera within the Characiformes order [14]. Recently, based on phylogenetic analysis performed using Cytochrome C Oxidase subunit I (COI) sequences, Rossini et al. [15] separated the *Astyanax* genus into five clades, with Clade 1 including *A. scabripinnis*, *A. paranae*, *A. bockmanni*, and *A. fasciatus*. Among them, the first two belong to the *A. scabripinnis* species complex [16]. Within the genus, 11 species carry B chromosomes of various sizes and morphologies, of which seven species belonging to the Clade 1 contain large metacentric B chromosomes [17–40] (Additional file 1: Table S1). This prevalence among species led Moreira-Filho et al. [39] to suggest the common origin of B chromosomes in several *Astyanax* species, and this has been supported by evidence from repetitive DNA content [41]. However, similarity in B chromosome morphology and size, chromosome banding, and satellite DNA location might simply be the result of convergent evolution instead of common descent.

The recent finding that B chromosomes can harbor protein-coding genes (for instance, see Navarro-Dominguez et al. [6]) provides a direct test for the common descent hypothesis, since the likelihood of finding the same gene content for B chromosomes of different species by chance is negligible. Here we analyze the protein-coding gene content of the large metacentric B chromosomes in *A. scabripinnis*, *A. paranae*, *A. bockmanni*, and *A. fasciatus* by comparing genomic and transcriptomic Illumina data between 1B (with B chromosomes) and 0B (without B chromosome) individuals for the two first sister species and by means of qPCR in the four species. We found that the large metacentric B chromosomes reported in these fish species most likely arose in a common ancestor at least 4 million years ago. In addition, the transcription of some cell cycle gene paralogs residing on these B chromosomes could potentially facilitate their drive during cell division. The extended permanence of B chromosomes in *Astyanax* species demonstrates the capacity of B chromosomes to resist population differentiation during speciation.

## Results

### Identifying B-linked genes

**Fig. 1:**
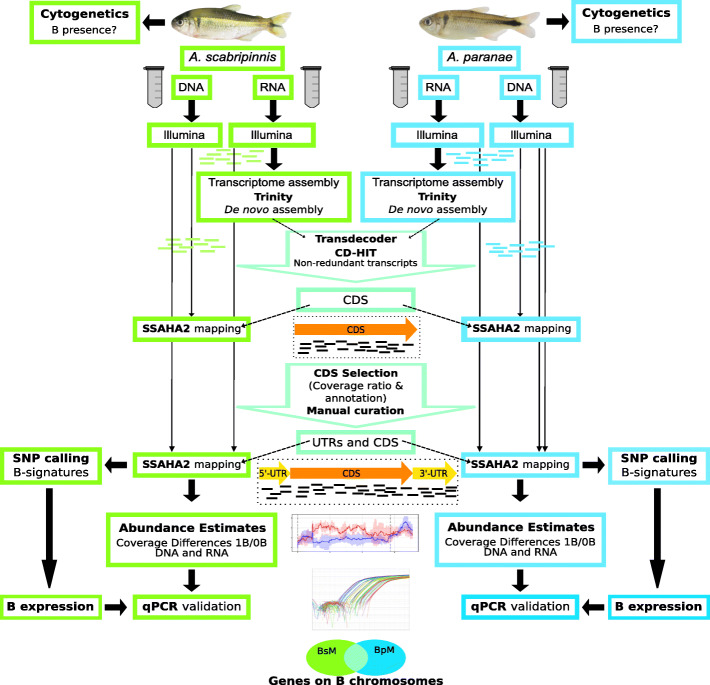
Bioinformatic protocol applied for the analysis of gene coverage, gene selection, and SNP calling. See Methods for details

**Fig. 2:**
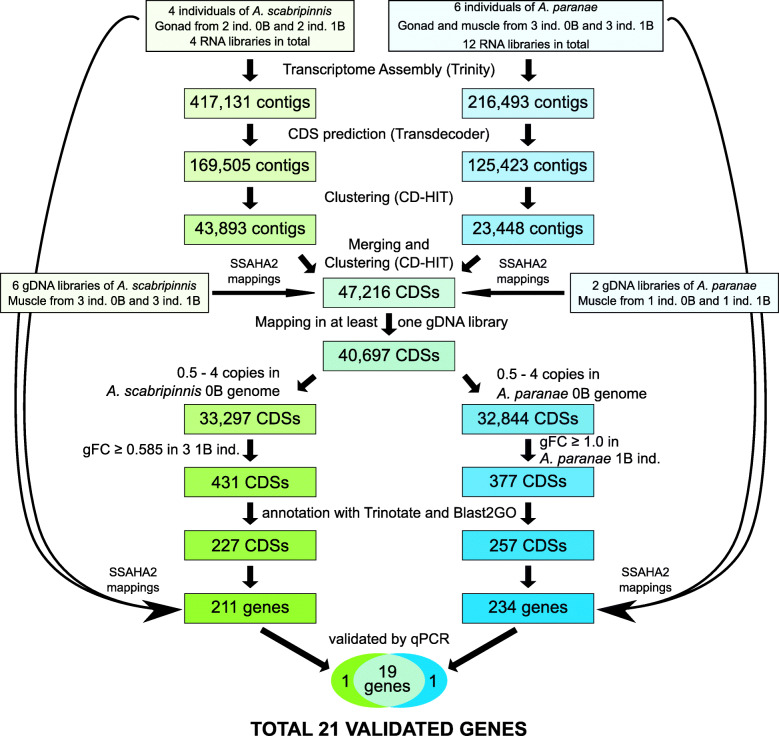
Pathway for gene search in the *A. scabripinnis* and *A. paranae* B chromosomes

We performed cytogenetic analyses prior to the Illumina sequencing and qPCR validation to determine the presence or absence of the large metacentric B chromosomes in the individuals of the four species analyzed here, as reported in previous studies (Table S1). The general pipeline of this work is summarized in Fig. 1. We first built a de novo transcriptome with a total of 16 female RNA libraries: four in *A. scabripinnis* (ovaries from two 0B and two 1B individuals) and 12 in *A. paranae* (ovaries and muscle from three 0B and three 1B individuals). We used this de novo transcriptome assembly as a reference for mapping all gDNA and RNA libraries to quantify coverage differences between B-lacking and B-carrying genomes. After mapping and copy number calculation, a total of 40,697 transcriptome contigs showed mappings of gDNA reads on them. Then we discarded all contigs showing more than four genomic copies (as they might be highly repetitive elements) and those showing less than 0.5 copies (since they might have had mapping errors) in the B-lacking genomes. The remaining 33,297 and 32,844 contigs showing mappings in *A. scabripinnis* and *A. paranae*, respectively, were then analyzed for overabundance in the B-carrying libraries with respect to the B-lacking ones. Assuming the presence of one gene copy per A or B chromosome, we calculated a theoretical minimum threshold that a transcriptome contig should pass to suggest the presence of one (or more) extra (paralogous) copy(s) on the B chromosome, in terms of the fold change in genomic coverage (gFC) attributable to B presence: gFC = log_2_ (1.5) ≥ 0.585, where 1.5 is the quotient between copy numbers in the 1B and 0B genomes (3 copies in 1B, 2 copies in 0B). In *A. scabripinnis*, we had gDNA libraries from three 0B and three 1B individuals, so that we calculated gFC for each B-carrying individual (by dividing its copy number by the average of the three B-lacking individuals) and selected those contigs showing gFC ≥ 0.585 in all three B-carrying individuals as candidates for being B-linked (genes present on B chromosome, but not exclusively) (Fig. 2, Additional file 2: Dataset 1). In *A. paranae*, however, we had Illumina libraries from only one B-carrying and one B-lacking individual, and thus increased the threshold to gFC ≥ 1.0 to limit the number of false positives.

**Fig. 3:**
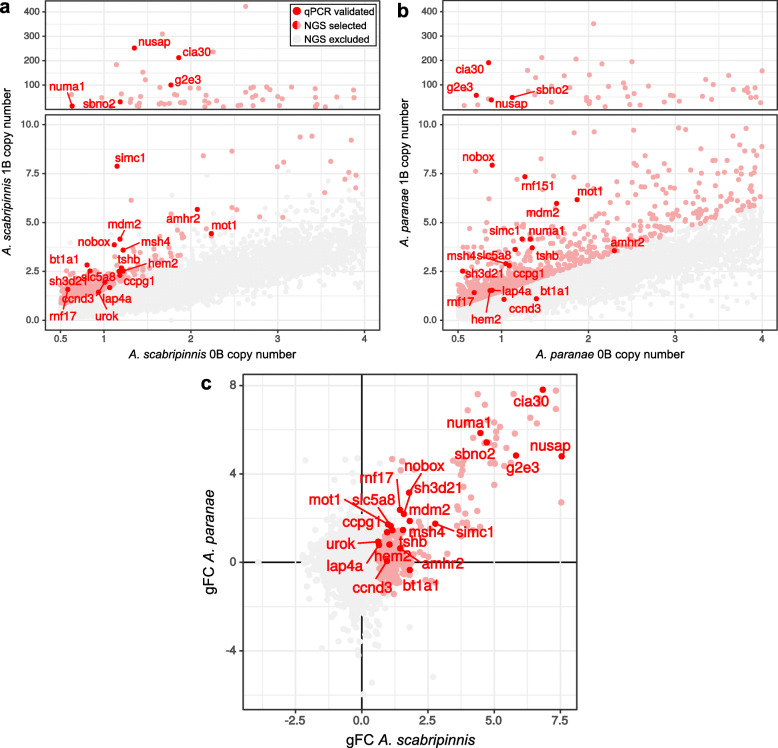
Detection of protein-coding genes residing in the B chromosomes of *Astyanax scabripinnis* and *A. paranae*. **a** Detection of protein-coding genes in the B chromosome of *Astyanax scabripinnis* by mapping gDNA Illumina reads obtained from three B-carrying (1B) and three B-lacking (0B) individuals on a de novo transcriptome built with Illumina reads coming from ovary RNA libraries from two individuals. Scatter plot showing the mean copy number for 47,216 CDSs in 0B and 1B females. The CDSs which, in presence of one B chromosome, showed a genomic fold change [gFC = log_2_(1B/0B)] higher than 0.585 in the three B-carrying individuals (431 contigs in total, see Supplementary Table 11) are shown in light red color, and those failing to reach this threshold are noted in gray color. In addition, the 21 protein-coding genes which were validated by qPCR in *A. scabripinnis* and *A. paranae* are noted in dark red color for the contig showing the highest gFC value. **b** Distribution of coverage in copy numbers for CDSs in 1B and 0B individual in *A. paranae*. Light red indicates the 2019 out of 32,830 CDSs with gFC in *A. paranae* higher than 0.585 and dark red indicated the 21 genes validated with qPCR. Note that only *bt1a1* and *ccnd3* genes do not meet the gFC criteria, but *bt1a1* was validated in these species using PCR. **c** Protein-coding gene content of the B chromosomes is highly similar among species. The figure shows the comparison of gFC values between *A. scabripinnis* and *A. paranae* for the CDS meeting the copy number criteria in *A. scabripinnis* 0B individuals. The gFC values found for the 21 B chromosome genes found in both species were positively correlated (Spearman rank correlation: *r*s = 0.27, *t* = 3.11, *P* = 0.002). Color patterns coincide with those in panel **a**

This analysis of gFC, performed on the Illumina B-carrying and B-lacking gDNA libraries, revealed 431 contigs showing gFC ≥ 0.585 in *A. scabripinnis* (Additional file 2: Dataset 1.5a) and 377 contigs showing gFC ≥ 1.0 in *A. paranae* (Additional file 2: Dataset 1.5b). Annotation revealed that 227 contigs being candidate to be B-linked in *A. scabripinnis* corresponded to 211 protein-coding genes, with some contigs belonging to the same gene. Similarly, in *A. paranae* 257 contigs corresponded to 234 protein-coding genes. Remarkably, 34 contigs corresponding to protein-coding genes found in the former species were also present in *A. paranae* (15%), a result that cannot be explained by chance, as shown by the binomial likelihood calculated for 15 coincidences and 85 differences (out of 100) with *p* = 1/25 for coincidence (since the haploid chromosome number is 25 in these species) and *q* = 24/25 for difference (*P* = 8.47 × 10^− 6^). Note that this probability was calculated for the percent coincidences to circumvent the spreadsheet problem to calculate factorial numbers higher than 170, and our sample consisted of 227 contigs. In addition, the correlation between gFC values for these 34 contigs in both species was highly positive (*r*s = 0.87, *t* = 9.92, *P* < 0.000001) indicating very high correspondence in their presence in B-carrying libraries of both species (Fig. 3, Additional file 2: Dataset 1.6).

**Fig. 4:**
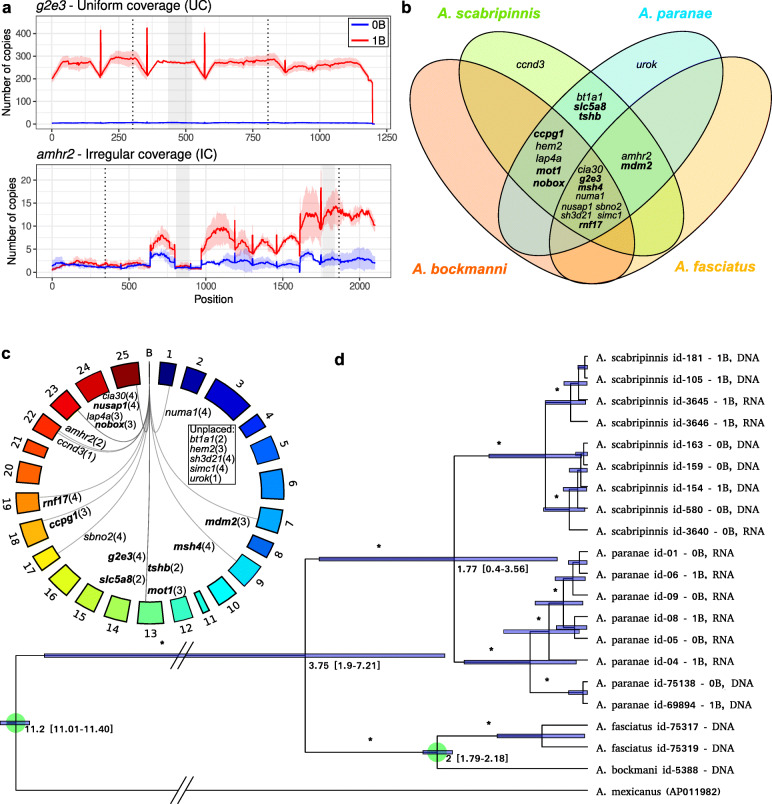
*Astyanax* B-linked genes sharing and B chromosome emergence. **a** Examples of the two coverage patterns found. Regions amplified by qPCR are shaded in gray. UC = uniform coverage and IC = irregular coverage. **b** Venn diagram showing the B-linked genes shared by the four *Astyanax* species analyzed here. Names in bold refer to genes with uniform coverage in the B chromosome of *A. scabripinnis*. Note that the approach used in this study cannot find unique genes in the lineages of *A. bockmanni* and *A fasciatus*. **c** Circos plot showing the location of the paralogs of B-linked genes on the regular chromosomes of *A. mexicanus*. Note that some validated genes were unplaced in the *A. mexicanus* genome. Names in bold refer to genes with uniform coverage in the B chromosome of *A. scabripinnis* and the numbers within parenthesis indicate the number of species where B chromosomes harbors the gene paralog. **d** Phylogram built with the full mitogenomes of all samples analyzed here. Green circles indicate the nodes used to calibrate the molecular clock. The most relevant result is that the node separating the four species carrying large metacentric B chromosomes is 3.75 mya. Intervals in node dates represent the 95% confidence interval. * = Bayesian posterior probability higher than 0.9

We then chose 27 candidate B-linked genes for qPCR validation (see the criteria in Methods) with primers designed in exons of the gene with high coverage in the 1B individuals comparing the 0B ones (Fig. 4a). This qPCR analysis revealed that 21 genes showed higher abundance in 1B gDNA libraries, of which 19 genes were higher in 1B individuals of both species (Table 1, Additional file 1: Table S2). The six remaining genes failed to show overabundance in 1B individuals from either species (Additional file 1: Table S3). Of note, the paired mean differences in the relative quantification (RQ) effect size due to B presence were strongly positively correlated between species (Spearman *r*s = 0.934, *t* = 13.08, *P* < 0.000001), supporting a high similarity in quantitative overabundance due to B presence in both species. We then searched for our 21 B-linked genes on the B chromosomes of two additional *Astyanax* species (*A. bockmanni* and *A. fasciatus*) to test the hypothesis of their common descent, comparing copy number between 1B and 0B individuals via qPCR on gDNA. The Gardner-Altman plot test [42] indicated that 14 of these genes in *A. bockmanni* and 11 in *A. fasciatus* are also B-linked (Fig. 4b, Additional file 1: Table S3).

**Table 1:** Identification, validation, and function of 21 protein-coding genes in the B chromosomes from the studied species

	*A. scabripinnis*			*A. paranae*		qPCR		
Gene	gFC	qPCR	SNPs	gFC	qPCR	*A. bockmanni*	*A. fasciatus*	Function
*amhr2*	1.8	1	7	0.9	1	0	1	Gonadal development
*bt1a1*	1.7	1	0	− 0.4	1	0	0	Unknown
*ccnd3*	1.2	1	0	−0.1	0	0	0	Cell cycle
*ccpg1*	0.9	1	9	1.3	1	1	0	Cell cycle
*cia30*	7.0	1	0	7.9	1	1	1	Mitochondrial
*g2e3*	5.7	1	0	5.0	1	1	1	Cell cycle
*hem2*	1.8	1	3	0.9	1	1	0	Biosynthesis
*lap4a*	1.5	1	0	1.1	1	1	0	Transport
*mdm2*	2.0	1	0	1.9	1	0	1	Indirect cell cycle regulation
*mot1*	1.0	1	1	1.6	1	1	0	Transport
*msh4*	1.6	1	21	1.4	1	1	1	Cell cycle
*nobox*	1.7	1	5	3.1	1	1	0	Cell cycle
*numa1*	2.7	1	0	2.5	1	1	1	Cell cycle
*nusap1*	7.3	1	0	3.9	1	1	1	Cell cycle
*rnf17*	1.3	1	2	1.9	1	1	1	Indirect cell cycle regulation
*sbno2*	5.9	1	0	6.8	1	1	1	Transcriptional coregulator
*sh3d21*	1.5	1	34	1.6	1	1	1	Unknown
*simc1*	3.1	1	25	2.0	1	1	1	Transcription factor
*slc5a8*	1.0	1	5	1.6	1	0	0	Transport
*tshb*	0.7	1	2	1.7	1	0	0	Hormone-mediated signaling pathway
*urok*	0.4	0	0	2.3	1	0	0	Plasminogen activation
Sum		20	114		20	14	11	

NGS analyses were performed for the large metacentric of *A. scabripinnis* and *A. paranae*, and, additionally, qPCR tests for their presence on B chromosomes in *A. bockmanni* and *A. fasciatus*. Genomic fold change (gFC) was calculated as log_2_(1B/0B) of mean nucleotide coverage along transcript length in the 1B and 0B libraries. In the qPCR columns, 1 indicates qPCR validation of gene overabundance in 1B genomes compared to 0B ones

Most qPCR-validated genes showed high gFC values, whereas most genes failing it showed low ones (see Table 1). Specifically, 17 out of the 18 genes showing gFC ≥ 1 in *A. paranae* were qPCR validated, as well as three genes (*amhr2*, *bt1a1* and *hem2*) failing to meet this condition. In fact, the gFC of *amhr2* and *hem2* were about 0.9, thus being close to the threshold employed in this species (gFC ≥ 1) (Table 1). In *A. scabripinnis*, the 20 genes that were qPCR-validated showed gFC ≥ 0.585 in all three B-carrying individuals analyzed, but no gene that failed to meet this condition was validated. These results indicate that gFC analysis is highly useful to perform efficient search for B chromosome genes and that using more biological replicates increases the efficiency of the B-linked gene selection method based on gFC. As a negative control, we also tried to qPCR validate the low coverage (LC) regions in four genes showing the IC pattern (*amhr2*, *numa1*, *sbno2*, and *simc1*). In *A. scabripinnis*, *A. paranae*, and *A. fasciatus*, all four genes failed to show overabundance for the LC region in B-carrying individuals (Additional file 1: Table S3). In *A. bockmanni*, however, the LC regions of the *amhr2* and *sbno2* genes also showed higher coverage in B-carrying individuals, indicating some differences in gene coverage patterns between the B chromosomes in these species.

### Non-random coincidences in gene content between species

Given that *Astyanax* genomes contain about 25 chromosome pairs, the probability for a given gene to be contained in an extra chromosome is 1/25 = 0.04, assuming that all chromosomes are of equal size (which clearly is not true, although for the average genome this may be a valid assumption). Using this estimate, we found that the binomial likelihood that B chromosomes in *A. paranae*, *A. bockmanni*, and *A. fasciatus* would carry, by chance, 19, 14, or 11 protein-coding genes, respectively, out of the 21 found in the B chromosome of *A. scabripinnis*, was extremely low (5.3 × 10^− 25^, 2.3 × 10^− 15^, and 9.8 × 10^− 11^). Therefore, the gene content similarity between the B chromosomes of these four species cannot be explained by chance.

### Origin of the B chromosome

We investigated whether B chromosomes in the four species analyzed here were inherited from a common ancestor species. Under this hypothesis, we should expect that the overlap in gene content between B chromosomes in the four *Astyanax* species is inversely proportional to the time since they shared their last common ancestor. To test this hypothesis, we assembled the full mitogenome sequence in the four species and built a phylogenetic tree using the *A. mexicanus* mitogenome as outgroup (Fig. 4d). We then performed a cluster analysis with the binary information of gene content overlap between the four species (see Additional file 1: Table S3) and extracted the matrix of Euclidean distances. Finally, a Mantel test showed a highly positive correlation between the matrices of *p* distances for mitogenome sequences and Euclidean distances for B chromosome gene content (*r* = 0.865, *P* = 0.043, based on 10,000 replicates). This indicated that B chromosome gene content is consistent with the phylogenetic relationships of these species, strongly suggesting that the B chromosome arose in a common ancestor of all four species, roughly 4 mya.

It is important to note that our results could also be compatible with recurrent B chromosome emergence in different species. As recent research has revealed that B chromosomes appear to be composed of DNA sequences acquired from many different A chromosomes [3], we investigated this possibility by mapping the 21 B-linked genes on the *A. mexicanus* A chromosomes (NCBI Genome assembly accession number GCA_000372685.2 [43]). We found 16 of these genes located on ten different chromosomes (Fig. 4c), making it unlikely that B chromosomes would have received the same genes from the same A chromosomes independently in the four species. Moreover, it was remarkable that incomplete genes missed exactly the same regions in different species, a fact that would be more parsimoniously explained through common descent (Additional file 1: Table S3).

### Expression of B-linked genes

We next identified B-specific SNPs for the 19 B-linked genes found in both *A. scabripinnis* and *A. paranae* (Additional file 1: Table S4), which we designated as *Alt* (alternative sequence variants) to differentiate them from the reference (*Ref*) variants found in the 0B libraries. We found 114 *Alt* variants in 11 genes, 106 of which showed counts in both species (Additional file 1: Tables S4, S5).

**Fig. 5:**
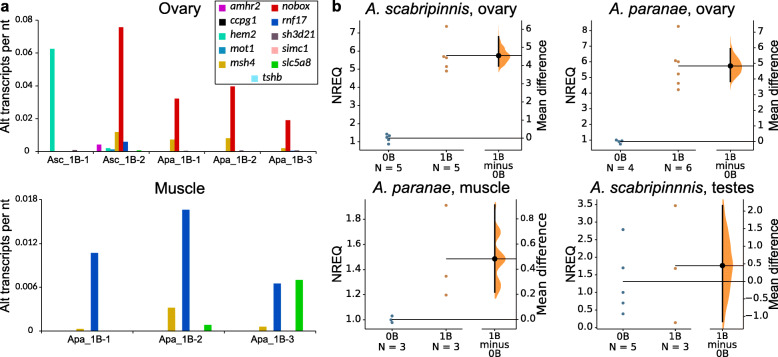
Transcription of B-linked genes in *Astyanax* species. **a** Gene expression profile of the B chromosomes of *A. scabripinnis* (Asc) and *A. paranae* (Apa) in gonadal and somatic tissues. Note that most B-linked transcripts belong to the *nobox* gene in the ovary. **b** Gardner-Altman estimation plots showing *nobox* transcription level in 0B and 1B individuals, analyzed by RT-qPCR. Both groups are plotted on the left axes, and the mean difference (effect size) is plotted on a floating axis on the right as a bootstrap sampling distribution. The mean difference is depicted as a black dot, and the 95% confidence interval is indicated by the ends of the vertical error bar. Note that (i) effect size was almost 5-fold higher in B-carrying (1B) than B-lacking (0B) ovaries of *A. scabripinnis* and *A. paranae*, (ii) that it was one order of magnitude lower in muscle, and (iii) that there was no difference in testes (here displayed only for *A. scabripinnis*). NREQ = normalized relative expression quantity. Asc = *A. scabripinnis*. Apa = *A. paranae*

We also performed differential expression analysis in *A. scabripinnis* (ovaries from two 0B and two 1B females) and *A. paranae* (ovaries and muscle from three 0B and three 1B females). Transcriptome analysis of these 16 RNA libraries showed that most B paralogs displayed very low expression levels in the ovary, the only exception being the *nobox* gene which displayed 7.5–13.2-fold higher expression for the B-linked than for the A-linked paralog in *A. paranae*, and 30.8-fold higher expression in one of the two females analyzed in *A. scabripinnis* (Additional file 1: Table S6). In muscle cells from the same *A. paranae* females, however, the *nobox* gene was inactive, as expected for a gene involved in oogenesis regulation. Intriguingly, the *msh4* gene showed 5.5-fold higher expression of the B-linked paralog in muscle, but it was observed in only one of the three individuals analyzed. Likewise, in *A. scabripinnis*, one of the two females analyzed showed no *nobox* transcription, presumably due to age differences between the two females analyzed (Fig. 5a, Additional file 1: Table S6). These results indicate that most B-linked genes were transcriptionally inactive in the individuals analyzed, although the strength of this conclusion is limited to the tissues, developmental stages, and environmental conditions under which our present experiments were performed. Additional RT-qPCR experiments for the *nobox* gene in both species showed about 5-fold effect size (mean differences) for transcription level in B-carrying ovaries compared to B-lacking ones, only 0.5-fold difference in female muscle, and no difference at all in testes (Fig. 5b, Additional file 3: Dataset 2). This result confirmed the upregulation of the *nobox* gene in the ovaries of B-carrying females, which, as shown above, is due to the active transcription of the B-chromosomal paralogs.

Among the four IC genes, *numa1* and *sbno2* did not exhibit B-specific SNPs and we could not analyze B-specific expression for them (Additional file 1: Table S4) although the gene coverage profiles in B-carrying individuals indicated that the HC regions showed higher transcription than LC ones (Additional file 4: Dataset 3). In addition, the *simc1* gene showed B-specific SNPs but we found no expression for it (Additional file 1: Table S4), suggesting its silencing in the life cycle stages analyzed. Finally, the B-specific SNPs found in the *amhr2* gene allowed detecting its expression in the ovary of one *A. scabripinnis* individual, suggesting that it might be a processed pseudogene (Additional file 1: Table S4).

## Discussion

The similarity in gene content for B chromosomes in four *Astyanax* species and the positive correlation between the matrices of *p* distances (for mitogenome sequences) and Euclidean distances for gene content suggest their common descent. The lower number of genes shared by the B chromosomes in *A. bockmani* and *A. fasciatus* might be due either to their actual absence in the B chromosome of these species, or to the accumulation of sequence differences in the gene regions where the qPCR primers were designed to anchor (which were conserved regions in *A. scabripinnis* and *A. paranae*), because of which the primers might have not worked in *A. bockmani* and *A. fasciatus*. Therefore, the absence of qPCR amplification for certain genes in *A. bockmanni* and *A. fasciatus* does not allow to rule out the presence of these genes on their B chromosomes, as the DNA sequences on which the PCR primers should have anchored might have changed. In any case, however, the observed differences in the qPCR results make it unlikely that the B chromosomes of the four species are the result of recent interspecific hybridization, but we cannot still rule out this possibility for the sister species *A. scabripinnis* and *A. paranae*. An example of a supernumerary chromosome arisen through experimental interspecific hybridization was reported by Perfectti and Werren [44] in the parasitic wasps *Nasonia vitripennis* and *N. giraulti*. In fact, the paternal sex ratio (PSR) chromosome in *N. vitripennis* contains DNA sequences showing higher similarity with those in wasps genus *Trichomalopsis* than with the A chromosomes from *N. vitripennis* [7, 45], suggesting its origin through interspecific hybridization. Interspecifically arisen B chromosomes have also been reported in the gynogenetic fish *Poecilia formosa* [46], and possible horizontal transfer of B chromosomes between species has also been suggested for the bee genus *Partamona* [47].

In the case of the four *Astyanax* species analyzed here, the high similarity in B chromosome gene content cannot be parsimoniously explained by chance or independent origin, whereas the observed differences make recent hybridization unlikely. Therefore, we believe that the most parsimonious explanation is that the B chromosomes in these four species were inherited from a B that was already present in a common ancestor. Among the genes validated by qPCR, the higher number of coincidences in B-linked gene content between the sister species *A. scabripinnis* and *A. paranae*, and the lower figures observed in *A. bockmanni* and *A. fasciatus*, are inversely proportional to the time since they shared their last common ancestor. The phylogenetic tree built with full mitogenomes indicated that the large metacentric B chromosome found in the four species analyzed here arose about 4 mya, i.e., since they split from their common ancestor. Even though our results are consistent with the shared origin of the B chromosomes in the four species, we admit that our experimental design is not ideal, since our reference transcriptome was assembled using the sister species *A. scabripinnis* and *A. paranae* but not included transcriptome or genome analyses in *A. bockmanni* and *A. fasciatus*. The absence of this information precluded species-specific primer design and thus we cannot be sure whether negative qPCR results for some genes in these two species are due to gene absence in their B chromosomes or, as commented above, to sequence divergence in the gene regions were primers were designed to be anchored. A recent study has reported the gene content of B chromosomes in two more distantly related *Astyanax* species, namely *A. correntinus* and *A. mexicanus* [21], but the absence of coincidence with the four species studied here suggests an independent origin for B chromosomes in these two species.

The long-term persistence of B chromosomes in *Astyanax* species demonstrates that B chromosomes can endure episodes of population divergence leading to species formation, even though their number is not fixed to one per haploid genome (as standard chromosomes) and they do not have regular meiosis. Our results also indicate that genomes are able to face major evolutionary challenges, such as speciation, in spite of bearing these extra chromosomes. This is also supported by recent research suggesting that all passerine birds carry a germline-restricted chromosome which likely descended from a B chromosome (GRC) [11, 48].

Eight out of the 21 B-linked genes detected here code for functions related to cell cycle regulation and gametogenesis (see Table 1). If active and functional in the right place and time, these eight genes might influence crucial processes for B chromosome transmission, such as mitosis or meiosis. Unfortunately, B chromosome transmission has not yet experimentally been analyzed in any *Astyanax* species. Two fish species where this has been done gave different results, suggesting the need for case-by-case analysis. No drive was found in *Prochilodus lineatus* [49–51] whereas micro-B-chromosomes in gynogenetic *Poecilia formosa* were paternally transmitted through genetic leakage [52]. In addition, Clark and Kocher [53] demonstrated that the B chromosome of the cichlid fish *Metriaclima lombardoi* exhibits drive, with 70% average transmission rate, because it carries a female sex determiner. Therefore, although it is conceivable that B chromosomes in *Astyanax* needed to show some kind of drive for successful passage through several speciation events, its real existence remains a mystery.

A good indication of the potential functional importance of B-linked genes is the degree at which they are transcribed. Based on B-specific sequence signatures, our transcriptome analysis showed that most B chromosome transcripts from the B-linked genes confirmed by qPCR belong to genes with functions related to cell cycle regulation, as would be expected for a parasitic B chromosome trying to manipulate cell divisions. In *A. scabripinnis*, one of the two 1B females showed a completely different transcriptional profile compared with those in the other females analyzed here, due to the fact that it was the only sampled immature female (Fig. 5a). The other female, however, showed a transcriptional profile very similar to that observed in the three 1B *A. paranae* females, where about 90% of transcripts coming from the B chromosome belonged to two cell cycle genes (*msh4* and *nobox*), and more than 85% of them corresponded to *nobox*. Bearing in mind that these two genes represented only 18% of the 11 genes showing B-specific sequence signatures, it is clear that the transcriptional profile of B chromosomes is highly biased towards functions dealing with cell division control. It was remarkable that the most actively transcribed B-linked genes showed a full transcript whereas those mostly silenced showed only a partial transcript, suggesting their pseudogenic nature.

Among the transcribed B-linked genes, it was noteworthy that (i) more than 70% of B-transcripts belonged to a single gene showing a functional role in oogenesis (*nobox*) in both species (Table S6); (ii) this was found in ovary but not in muscle RNA (Fig. 5); and (iii) for this gene, B-carrying individuals showed more intense transcription for B-linked paralogs than for the standard gene copies residing on the A chromosomes (almost 30-fold higher in *A. scabripinnis* and 7–13-fold in *A. paranae*) (Table S6). We are thus tempted to speculate that B chromosomes in *Astyanax* are playing their destiny during oogenesis, like B chromosomes in many other organisms. Whether this implies the existence of B chromosome drive during female meiosis needs to be clarified by means of inheritance analysis through controlled crosses, a task remaining for future research.

The observed upregulation of the *nobox* gene might provide a mechanistic explanation to previous results showing an association between B chromosome presence in *A. scabripinnis* and sex ratio distortion [54], hermaphroditism [55], or upregulation of the *dmrt1* gene [56]. The transcriptional profile of B-linked genes suggests that their expression might affect gonadal development, and in this context, it should also be investigated the possibility that pseudogenic B-transcripts for the *amhr2* gene might interfere this process, as this gene has been shown to be a master sex determining in other teleost fish [57]. The analysis of co-expression between the most highly transcribed B-linked gene (*nobox*) and other genes being crucial for sex and gonadal development (e.g., *dmrt1*) may help to clarify whether B chromosome presence in this species influences sex determination, as suggested in cichlid fishes [58], and to elucidate under which conditions B-chromosomal effects are apparent. The limitation of B-chromosomal effects to *dmrt1* expression in the testis, with no effects in the ovary [56], indicates that other genes, in addition to *nobox*, might be involved in these effects and/or B chromosome drive. For instance, the 5-fold transcription of the *Alt* sequence variant for the *msh4* gene (i.e., a pleiotropic gene involved in oogenesis and spermatogenesis), observed in muscle RNA from one *A. paranae* B-carrying individual, suggests that B-linked genes might play a relevant role in other life cycle stages, apart from oogenesis. On this basis, the apparent absence of the *nobox* gene in the B chromosome of *A. fasciatus* might also suggest the importance of other B-linked genes (e.g., *msh4*) as potential granters of B chromosome drive, which represents another interesting question to approach in future research.

## Conclusions

In summary, we show here that the similarity in gene content of B chromosomes in four species of fish of the genus *Astyanax* is correlated with these species’ phylogeny and is thus consistent with B chromosome presence in their most recent common ancestor which lived 4 mya. Thus, we demonstrated that B chromosomes can endure through speciation events despite lacking regular pairing and segregation during meiosis. The recent finding that a B-linked gene in *Nasonia vitripennis* is responsible for B drive [7] inspires the speculation that B-linked genes in *Astyanax*, such as *nobox*, might have helped B chromosome maintenance for the long timescales shown here. Likewise, as B-chromosomal genes are not fully silenced, the possibility exists that B-derived transcripts provoked gene expression changes affecting the species diversification of this genus.

## Methods

### Sampling and determination of B chromosome number

We analyzed samples from natural populations of *A. paranae*, *A. bockmanni*, *A. fasciatus*, and *A. scabripinnis* (Additional file 1: Table S7). Previous studies showed the presence of B chromosomes in all these populations [20, 27, 59, 60]. The animals were collected in accordance with Brazilian environmental protection legislation (Collection Permission MMA/IBAMA/SISBIO-number 3245) and the procedures for fish sampling, maintenance, and analysis were performed in compliance with the Brazilian College of Animal Experimentation (COBEA) and approved (protocol 504) by the BIOSCIENCE INSTITUTE/UNESP ETHICS COMMITTEE ON THE USE OF ANIMALS (CEUA). The specimens were identified and deposited at the fish collection of the Fish Biology and Genetics Laboratory, Botucatu, São Paulo, Brazil, under the vouchers LBP19572 (*A. paranae*) and LBP19573 (*A. fasciatus*). The specimens of *A. scabripinnis* and *A. bockmanni* were deposited at the Zoology Museum of the State University of Londrina, Londrina, Paraná, Brazil, under the voucher MZUEL 8371 and in the fish collection of Fish Genetics Laboratory, Bauru, São Paulo, Brazil, respectively.

B chromosome number of all individuals analyzed in this study was determined through cytogenetic analysis following the protocol described by Foresti et al. [61]. Briefly, the animals were treated with an aqueous solution of colchicine 0.025% during 40 min, anesthetized, and killed, and the chromosomal preparations were obtained from cells of the anterior kidney and gills. C-banding was performed according to the protocol described by Sumner [62], to improve accuracy in identifying the presence and number of B chromosomes in the samples. Chromosome preparations were stained for 5 min with 5% Giemsa solution in phosphate buffer (pH = 6.7). At least ten mitotic metaphase cells were analyzed per specimen. The images were captured with a digital camera (Olympus DP80) attached to an Olympus BX61 epifluorescence photomicroscope, using Image Pro Plus 6.0 software (Media Cybernetics). Image treatment, including optimization of brightness and contrast, was performed using the Adobe Photoshop CS4 program.

### Nucleic acid extraction and Illumina sequencing

We obtained genomic DNA (gDNA) from samples (Additional file 1: Table S8) using the NucleoSpin® Tissue Kit (Macherey Nagel) following the manufacturer’s instructions. We included a step for RNA removal by adding 20 μL of RNAse A (10 mg/mL) (Invitrogen) per sample and incubating for 5 min at room temperature. Samples were run on 1% agarose gel to check DNA integrity and then used for qPCR and Illumina sequencing experiments. For qPCR, we used DNA from muscle from *A. scabripinnis*, and liver from *A. paranae*, *A. bockmani*, and *A. fasciatus* species (Additional file 1: Table S8). No heteromorphic sex chromosomes have been reported in the extensive cytogenetic literature of *Astyanax* species, nor did our analyses reveal the presence of any heteromorphic bivalent in meiosis. Therefore, in absence of information on the possible existence of homomorphic sex chromosomes in these species, the only observable variation in chromosome number, between individuals of any *Astyanax* species, was due to the presence of B chromosomes. For this reason, we used males and females indistinctly, and muscle or liver for qPCR and ovary, testis, and muscle for RT-qPCR experiments (Additional file 1: Table S8). For Illumina sequencing in *A. scabripinnis*, we extracted genomic DNA from the muscles of three 0B individuals (one female and two males) and three 1B individuals (two females and one male), and they were sequenced using the platform Illumina HiSeq X Ten yielding read lengths of 2 × 150 bp. In *A. paranae*, however, we extracted genomic DNA from the liver of a 0B female and a 1B female, using Illumina HiSeq2000 with yielded read length of 2 × 101 bp (Additional file 1: Table S9). These differences between the Illumina platforms were due to their availability when we obtained them. Again, we expect that this has no substantial effect on the final results, since experimental design is homogeneous within every species and we performed the 1B and 0B comparison separately in every species.

In each species, the individuals used for RNA extraction were collected on the same day and were kept in a common aquarium. After dissection, tissues were immediately frozen in liquid nitrogen and stored at − 70 °C. The analyzed tissues are described in Additional file 1: Table S8. RNA was extracted using the TRIzol® Kit (Invitrogen), following the manufacturer’s instructions. Subsequently, the samples were treated with DNaseI (Thermo Fisher Scientific) and checked on a 1% agarose gel and on a 2100 Bioanalyzer® (Agilent) equipment. Only RNA samples with A260/280 ratio of 1.8–2.0, A260/230 ratio > 2.0, and RIN > 7 were used for subsequent analysis. Here we used female RNA for RT-qPCR in *A. scabripinnis* and *A. paranae* species (Additional file 1: Table S8). For Illumina sequencing, we used two 0B ovary and two 1B ovary in *A. scabripinnis* and three individuals for 0B and 1B ovary, and 0B and 1B muscle in *A. paranae* (Additional file 1: Table S9). We focused our expression analysis on the two species where B chromosomes are most easily found, as samples with different number of B chromosomes should be obtained under the same environmental conditions in order to avoid gene expression changes due to spatial or temporal differences thus obscuring the differences due to B chromosome presence. In the two other species, the B chromosomes are in a low proportion of individuals in the natural populations, and to obtain several individuals with B chromosomes requires a great effort and several days of field sampling. The differences in the number of samples were due to budget restrictions. In addition, in *A. paranae* we decided to obtain samples of a somatic tissue to compare gene expression with gonadal tissues and testing the hypothesis of higher B chromosome impact on gonadal tissues.

We newly sequenced all libraries for the present work, except for the gDNA samples in *A. paranae,* which were previously sequenced by Silva et al. [41] (Additional file 1: Table S9).

### Bioinformatic analysis

The bioinformatic protocol (Fig. 1) used to select candidate genes present in B chromosomes constitutes a new pipeline called whatGene [63]. Our custom scripts used in this study are freely available in a repository [64]. We first generated independent de novo assemblies of the transcriptomes in *A. scabripinnis* and *A. paranae*, separately, using all the RNA-seq libraries, i.e., four and twelve, respectively (Additional file 1: Table S9). We assembled them using the Trinity software v2.1.1 [65], with default options for trimming, in silico normalization and assembly. Then we used Transdecoder v2.0.1 [65] to predict and extract the CDS from the contigs and removed redundancies using CD-HIT v4.6.4 [66] with options “-M 0 -aS 0.8 -c 0.8 -G 0 -g 1”. After that, we merged the resulting files from both species into a single reference transcriptome and removed redundancies again with CD-HIT using the same options. Finally, we proceeded to the annotation with Trinotate v3.2.0 (https://trinotate.github.io) and Blast2GO v5.2.5 [67]. Note that the main limitation in mapping genomic reads against a reference transcriptome is that coverage in exon junctions is a bit reduced. However, as we used software supporting partial mappings of the reads, it is not a big problem. Advantages of using a reference transcriptome in case of non-model species with large and unsequenced genomes are that (i) it is more easy to assemble than genomic sequences by lacking introns, (ii) the analysis is focused in the part of the genome with putatively functional roles, and (iii) the interference of repetitive elements on assembling is minimal, as we removed those transcripts showing up to four copies per haploid genome in 0B individuals.

The next step consisted in mapping gDNA reads against the reference transcriptome, using SSAHA2 v2.5.4 [68] with a mapping length of least 40 nt and with a minimum identity of 80% using the “ssaha2_run_multi.py” script [64]. We then converted CDS coverage to copy number per haploid genome, taking into account library size and that the diploid genome size of *A. scabripinnis* is 3.74 pg [69]. To calculate the genome size of 1B individuals, we estimated the relative size of the B chromosome on chromosome preparations as ~ 5% of A chromosomes per haploid genome (i.e., 0.093 pg), so the 1B genome size would be equal to 3.83 pg. We finally converted pg to Gb, considering that 1 pg of DNA corresponds to 0.978 Gb [70]. The resulting diploid genome sizes for 0B and 1B individuals were 3.658 and 3.749 Gb, respectively. We considered the same values in the case of *A. paranae*, since they are sister species belonging to the same species complex [16].

We extracted coverage information per nucleotide position for all mapped libraries, using the “bam_coverage_join.py” script [64], and then used the “coverage_graphics.py” script [64] with the “NOPLOT” option to estimate the copy number per haploid genome for every CDS. Next, we discarded, in each species, those contigs that presented less than 0.5 or more than 4 copies per haploid genome, to minimize cases of putative assembly problems or repetitive DNA, respectively. As 1B individuals carry two A chromosome sets plus one B chromosome, and assuming one gene copy per A chromosome and B chromosome, we calculated the genomic fold change (gFC) in copy number expected between 1B and 0B genomes as log2 of the 1B/0B quotient [i.e., log2(3/2) = 0.585]. In *A. scabripinnis*, we calculated gFC for each 1B individual separately, in reference to the average of copy numbers in the three 0B individuals, and then we performed a first contig selection including all those where gFC ≥ 0.585 in all three 1B individuals. As a single 1B individual was sampled for *A. paranae*, we conservatively selected those contigs with gFC ≥ 1. Then, we selected annotated contigs, excluding repetitive elements. For these mappings and calculations, we assumed that A and B chromosome gene copies are paralogs showing highly similar sequence, as previously reported in another B chromosome system [41], so that A- and B-derived reads can map against the same reference sequence.

Some of the selected CDSs in the reference transcriptome that passed this annotation filter were incomplete, as they lacked start and/or stop codons, whereas other CDSs belonged to different regions of the same gene but had been assembled in different contigs. We solved these assembly issues by performing manual curation to get the longest contigs as possible, in the first case, and a single contig per gene in the second case. For this purpose, we searched for their full sequence in the 0B de novo transcriptome assemblies generated for each species and also in the combined 0B and 1B libraries using BLASTN v2.10.0 [71] with default parameters. This strategy yielded a short collection of contigs (in most cases including UTR and CDS regions), which were used as a reference to perform new mappings of gDNA and RNA Illumina reads from different individuals, using SSAHA2 with the same options described above. We processed the resulting mappings by extracting the counts per position with the “bam_coverage_join.py” script [64] and then using the “coverage_graphics.py” script [64] with “PDF” option to get the average coverage per gene and generate coverage plots along every gene in *A. scabripinnis*. Visual inspection of these coverage plots showed the existence of two types of B-chromosomal genes, i.e., those showing coverage uniformly higher in 1B genomes (UC = uniform coverage) and those showing higher coverage only in some gene regions (IC = irregular coverage) (Fig. 4a, Additional file 4: Dataset 3). In IC genes, we identified regions showing high (HC) or low (LC) coverage delimited by abrupt coverage changes. This allowed estimating gene copy numbers separately for HC and LC regions for each IC gene, using the “coverage_graphics.py” script [64] with the “NOPLOT” option. HC and LC regions of IC genes where then chosen to perform separate qPCR analysis for genomic overabundance of some of these genes in B-carrying individuals.

We also searched for B-specific nucleotide variation in *A. scabripinnis* through SSAHA2 mapping of gDNA and RNA libraries against the manually curated contigs. For this purpose, we merged all mappings from 0B gDNA, 1B gDNA, 0B RNA, and 1B RNA (i.e., four mapping files in total), using SAMtools (v1.10) [72], and extracted the counts per position for the four nucleotides, including insertions and deletions, using the “bam_var_join.py” script [64]. Then we used the “snp_calling_bchr.py” script [64] to select those SNPs being fixed in the 0B gDNA (*Ref* allele), which showed counts for an alternative allele exclusive of 1B gDNA (*Alt* allele). Then we filtered out the SNPs showing a lower proportion of *Ref* than *Alt* alleles to minimize the frequency of *Ref* alleles in the B chromosome. Since it is not possible to distinguish between *Ref* alleles from A and B paralogs, we decided to remove SNPs with a higher proportion of *Ref* alleles respect to *Alt* alleles in 1B individuals. In practice, we found that this pattern occurs in those genes showing many copies on the B chromosome, where some B-linked copies have the *Ref* allele and other B-linked copies have the *Alt* allele. After that, we extracted the counts for the *Ref* and *Alt* alleles for the selected SNPs in every single library from *A. scabripinnis* and *A. paranae* (gDNA and RNA), first using the “bam_var_join.py” script [64] with the 24 libraries, and then the “get_var_library.py” script [64]. To enrich the SNP sample in those conserved between *A. scabripinnis* and *A. paranae* A chromosomes, we removed SNPs with two or more counts for the *Alt* allele in the 0B *A. paranae* gDNA or RNA. Finally, we considered multiple adjacent missing basepairs were considered as a single mutational event.

To investigate the possible transcription of the paralogous gene copies located on B chromosomes, we scored the number of reads showing the *Ref* and *Alt* variants for the 114 SNPs in each RNA Illumina library from both species (12 in *A. paranae* and 4 in *A. scabripinnis*). We calculated the quotient between the number of reads scored and transcript length, to normalize among genes. Finally, we calculated the intensity of B chromosome transcription for these genes as the *Alt*/*Ref* ratio in B-carrying individuals, as it indicates how many transcripts for a given gene came from the transcription of the paralog copy located on the B chromosome, compared with those coming from the A chromosomes.

We analyzed the chromosome location of B-linked genes in the *A. mexicanus* genome assembly (NCBI Assembly accession number GCA_000372685.2 [43]) and represented it in a Circos plot v0.69-9 [73]. We note that this reference would be valid only if assuming full synteny with the species sampled here.

### Quantitative real-time PCR (qPCR)

We tested the overabundance of B-linked genes in B-carrying individuals by means of qPCR on gDNA. For this, we selected 24 genes with gFC ≥ 0.585 on HC regions in all the three 1B individuals of *A. scabripinnis*. Then, we designed a single primer pair in genes showing the UC pattern, and two primer pairs (for the HC and LC regions) in case of IC genes with Primer3 software [74] (Additional file 1: Table S10). We also included three genes in which contigs did not meet all the criteria (*irl1b*, *lap4a*, *urok*) to explore the validity of the thresholds used (Additional file 2: Dataset 1.7).

Relative quantification (RQ) of the copy number for B chromosome genes was performed using the samples described in Additional file 1: Table S8 by quantitative PCR employing the 2^−∆Ct^ method [75]. Quantitative PCR was performed on QuantStudio™ 12K Flex Real-Time PCR Systems (Thermo Fisher Scientific, USA). The reactions were performed in a final volume of 10 μL, with 3 ng of gDNA, 5 μL of Power SYBR™ Green PCR Master Mix (Thermo Fisher Scientific, USA), and 1 μL of each 5 μM primer. Cycle conditions were 95 °C for 10 min; 45 cycles of 95 °C for 15 s, and 60 °C for 1 min. An autosomal single-copy gene, hypoxanthine phosphoribosyltransferase (*hprt*), was used as reference gene. Target and reference genes were analyzed simultaneously in triplicate for three independent samples. The specificity of the PCR products was confirmed by dissociation curve analysis.

In *A. bockmanni*, the gDNA of two 1B individuals was exhausted so that qPCR analysis could only be performed on two 1B individuals for nine genes and one 1B individual for six genes. In the latter case, we considered that these genes were in the B chromosome if the 1B individual showed RQ higher that all B-lacking individuals.

Quantification of expression levels of *nobox* gene was performed using the RNA samples described in Additional file 1: Table S8, using the same set of primers used for quantification of relative copy number. The primers were not designed to the B-specific sequences, so that they were able to amplify both A and B paralogs. The cDNA for each sample was synthesized using the High-Capacity cDNA Reverse Transcription Kit® (Thermo Fisher Scientific, USA) using 100 μg per sample of total RNA, following the manufacturer’s instructions. The cDNA obtained was diluted in RNase-DNase-free water for a 1:50 working solution. The RT-qPCR reactions followed the same thermocycler parameters used for the qPCR reactions, except for the use of cDNA solution instead of the gDNA. Target and reference genes were analyzed simultaneously in duplicates for two independent samples. The normalized relative expression quantity (NREQ) was determined by the 2^−∆∆Ct^ method [76], and *nobox* expression levels were normalized using *hprt* as the reference gene, with subsequent calibration to the average expression of the 0B group.

### Mitogenome assembly and phylogenetic analyses

We built a dated mitogenome phylogenetic tree to get an estimate of the age for the main lineages studied in this work. For this purpose, we included all the *A. scabripinnis* and *A. paranae* Illumina gDNA and RNA libraries used here, i.e., 24 libraries in total. We also used gDNA Illumina libraries from *A. fasciatus* and *A. bockmanni*, obtained by Utsunomia et al. (unpublished). We assembled the mitogenome for these 27 libraries using the NOVOPlasty software (v3.7) [77] using the *A. paranae* mitogenome (GenBank KX609386.1 [78]) as seed. We also included the *A. mexicanus* mitogenome (GenBank AP011982.1 [79]) as an outgroup. The sequences were aligned using the MAFFT software (v7.450) [80] with “auto” option and removed uninformative positions in the alignment with Gblocks (v0.19b) [81] to exclude the control region which is absent in the RNA libraries. Finally, we built a dated phylogenetic tree using BEAST (v1.10.4) [82]. As calibration points, we used the node separating *A. mexicanus* from the remaining species (11.2 mya) and the node separating *A. fasciatus* and *A. bockmanni* (2 mya), considering molecular dating in Piscor et al. [83]. We launched three independent runs with 100,000,000 generations and sampled trees every 1000 generations. Convergence was checked with Tracer (v1.7.1) [84] and burnin was set to 25%.

### Statistical analyses

Two-group comparisons were performed by the Gardner-Altman estimation plot method devised by Ho et al. [42] following Gardner and Altman’s design [85], as implemented in https://www.estimationstats.com/. Non-parametric correlation analysis was performed using Statistica 6.0 (Statsoft, Inc.).

## Supplementary Information

**Additional file 1: Table S1.** Diversity of B chromosome in the *Astyanax* genus. 2n = diploid chromosome number. Bs = range of B chromosomes found in each population. a = acrocentric. m = metacentric. sm = submetacentric. st = subtelocentric. *A. jordani* and *A. mexicanus* can be considered synonymous, since both references analyzed the blind tetra fish [24, 25]. **Table S2.** Statistics of 21 B-linked genes in *A. scabripinnis* and *A. paranae*. Genomic fold change (gFC) was calculated as the log_2_(1B/0B) quotient between the mean nucleotide coverages along transcript length. HC = High coverage region. LC = Low coverage region. IC = Irregular Coverage. UC = Uniform Coverage. Figures in the “ind. gFC ≥ 0.585” column indicates the number of 1B *A. scabripinnis* individuals which met this condition. In the “qPCR” columns, 1 indicates validation of gene presence on the B chromosome. **Table S3.** Validation of B-linked genes by qPCR. Genomic fold change (gFC) was calculated as the log_2_(1B/0B) quotient between the mean nucleotide coverages along transcript length. IC = Irregular Coverage. UC = Uniform Coverage. CI = confidence interval. The blank spaces indicate experiments not performed. In the “Binary information” columns, 1 means qPCR validation. Asc = *A. scabripinnis*. Apa = *A. paranae*. Abo = *A. bockmanni*. Afa = *A. fasciatus*. **Table S4.** Counts of reference (*Ref*) and alternative (*Alt* = B-specific) variants in all gDNA and RNA libraries analyzed in *A. scabripinnnis* and *A. paranae*. **Table S5.** Nucleotide variation found in the B chromosome gene paralogs of *A. scabripinnis*. To score the number of variants, we distinguished between substitutions (s), deletions (d) or insertions (i) with respect to the A chromosome paralogs. We considered deletions and insertions as a single mutation event when they involved two or more consecutive nucleotides. dS = number of synonymous substitutions. dN = number of non-synonymous substitutions. ps = proportion of variable sites. **Table S6.** Gene expression profile and transcription intensity of B chromosomes. See Methods for details. **Table S7.** Localities, diploid chromosome number (2n) and number of samples (N) of the *Astyanax* individuals analyzed cytogenetically in this study. All populations harbor a large metacentric B chromosome. **Table S8.** Description of the materials analyzed by quantitative PCR. **Table S9.** Description of the Illumina-sequenced materials. **Table S10.** Primers designed in this study for PCR amplification of B-linked genes.

**Additional file 2. **Dataset 1. Spreadsheet showing the procedure for CDS selection by applying consecutive filters: 1) All contigs showing mappings in gDNA libraries (40,679 contigs), 2a) Contigs with average number of copies, in the 0B libraries of *A. scabripinnis*, ranging between 0.5 and 4 (33,297 contigs), 2b) Contigs with average number of copies in the 0B libraries of *A. paranae* between 0.5 and 4 (32,844 contigs), 3a) Contigs showing genomic fold change due to B chromosome presence [gFC = log_2_(1B/0B)] higher than 0.585 in at least 1B individual of *A. scabripinnis* (1928 contigs), 3b) Contigs showing genomic fold change due to B chromosome presence [gFC = log_2_(1B/0B)] higher than 1 in the 1B individual of *A. paranae* (377 contigs), 4a) Contigs showing genomic fold change due to B chromosome presence [gFC = log_2_(1B/0B)] higher than 0.585 in all the three 1B individuals of *A. scabripinnis* (431 contigs), 5a) Contigs of *A. scabripinnis* annotated from *Astyanax mexicanus* genome excluding repetitive elements (227 contigs), 5b) Contigs of *A. paranae* annotated from *Astyanax mexicanus* genome excluding repetitive elements (257 contigs), 6) Combination of 5a and 5b data (450 contigs), 7) qPCR results associated with each contig. Note that the *irl1b* and *lap4a* contigs have gFC ≥ 0.585 in only two individuals of *A. scabripinnis*, while the *urok* gene meets this criterion in only one individual of this species, but the three genes have average gFC value about 0.65. Also note that the gFC of the *urok* gene in *A. paranae* is close to the threshold used.

**Additional file 3: **Dataset 2. Relative transcription levels for the *nobox* gene in B-carrying and B-lacking individuals. NREQ = Normalized relative expression quantity. (XLS 37 kb)

**Additional file 4: **Dataset 3. Coverage levels of the 21 protein-coding genes found in the *A. scabripinnis* and *A. paranae* B chromosomes. gDNA coverage is shown as number of copies. Additionally, we add a track showing the position of the B-specific SNPs found in the *A. scabripinnis* libraries. The dotted lines delimit the CDS region and the shaded zones indicate the regions amplified in the qPCR experiments. The coverage pattern attributed to each gene can be found in the Table 1.

## Data Availability

The Illumina libraries used for this article are available in the Sequence Read Archive (SRA) under Bioprojects PRJNA383714 [86], PRJNA560500 [87], PRJNA560501 [88] and PRJNA560502 [89] (see Additional file 1: Table S9 for details). Main data generated or analyzed during this study are included in this published article and its supplementary information files. The remaining datasets can be requested to the corresponding authors.
