# Supplementary material for: Long-term persistence of supernumerary B chromosomes in multiple species of Astyanax fish
Source: BMC Biol. 2021 Mar 19;19:52. doi: 10.1186/s12915-021-00991-9 (PMC7976721; doi:10.1186/s12915-021-00991-9)
Supplement: Supplementary file 4 — Additional file 4: Dataset 3. Coverage levels of the 21 protein-coding genes found in the A. scabripinnis and A. paranae B chromosomes. gDNA coverage is shown as number of copies. Additionally, we add a track showing the position of the B-specific SNPs found in the A. scabripinnis libraries. The dotted lines delimit the CDS region and the shaded zones indicate the regions amplified in the qPCR experiments. The coverage pattern attributed to each gene can be found in the Table 1. [file 12915_2021_991_MOESM4_ESM.pdf]

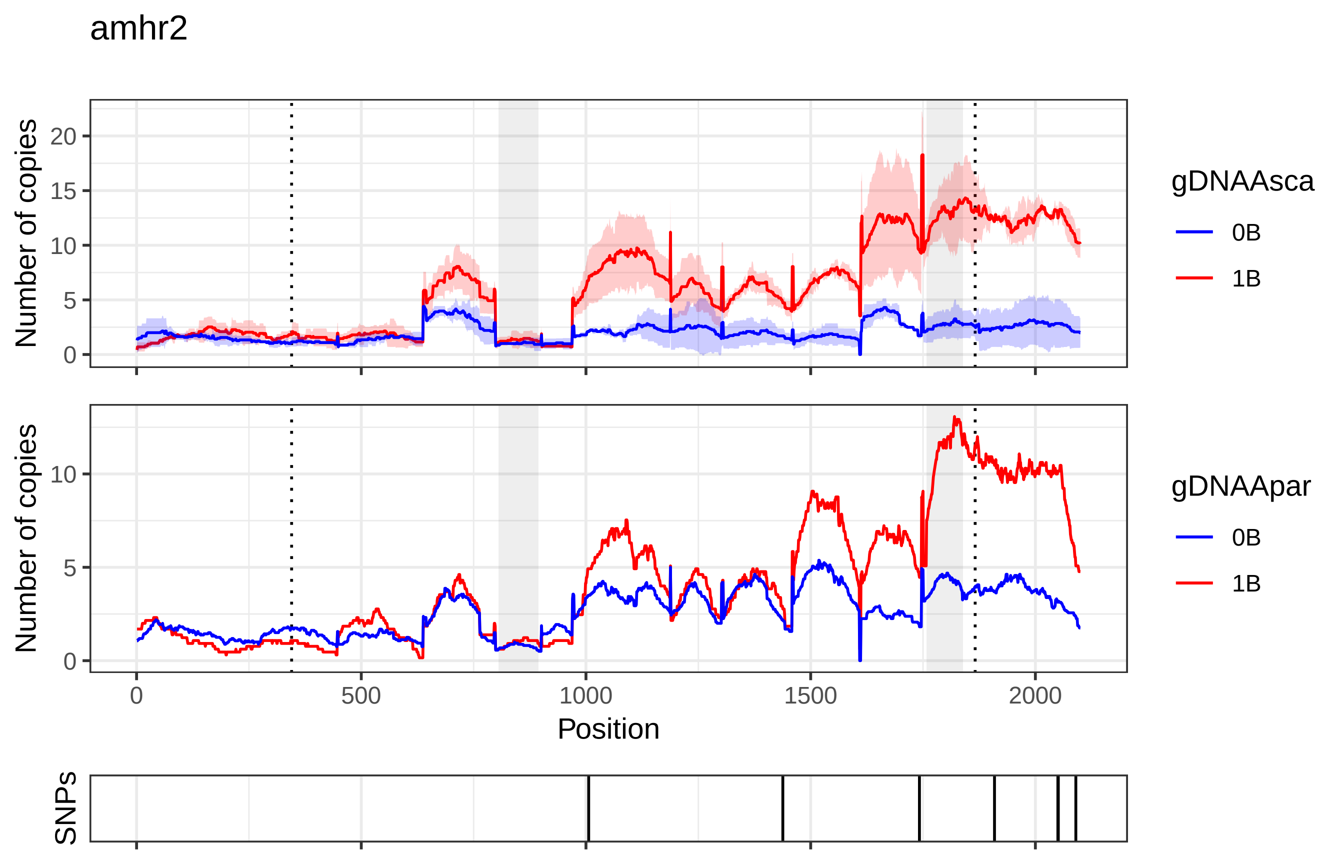
amhr2
Number of copies
20
15
10
5
0
gDNAAsca
0B
1B
Number of copies
10
5
0
gDNAApar
0B
1B
0
500
1000
1500
2000
Position
SNPs

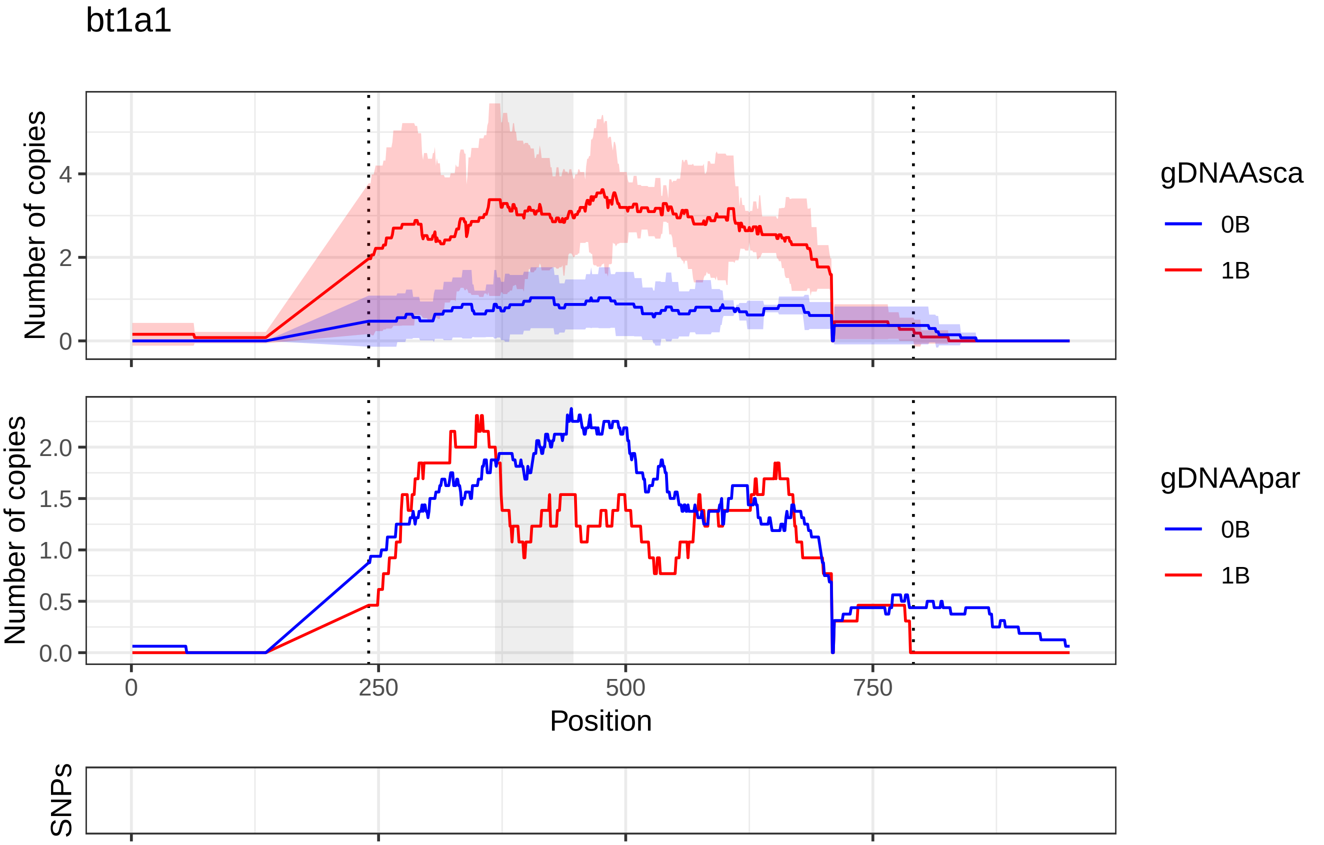
bt1a1
Number of copies
gDNAAsca
0B
1B
gDNAApar
0B
1B
0
2
4
0.0
0.5
1.0
1.5
2.0
0
250
500
750
Position
SNPs

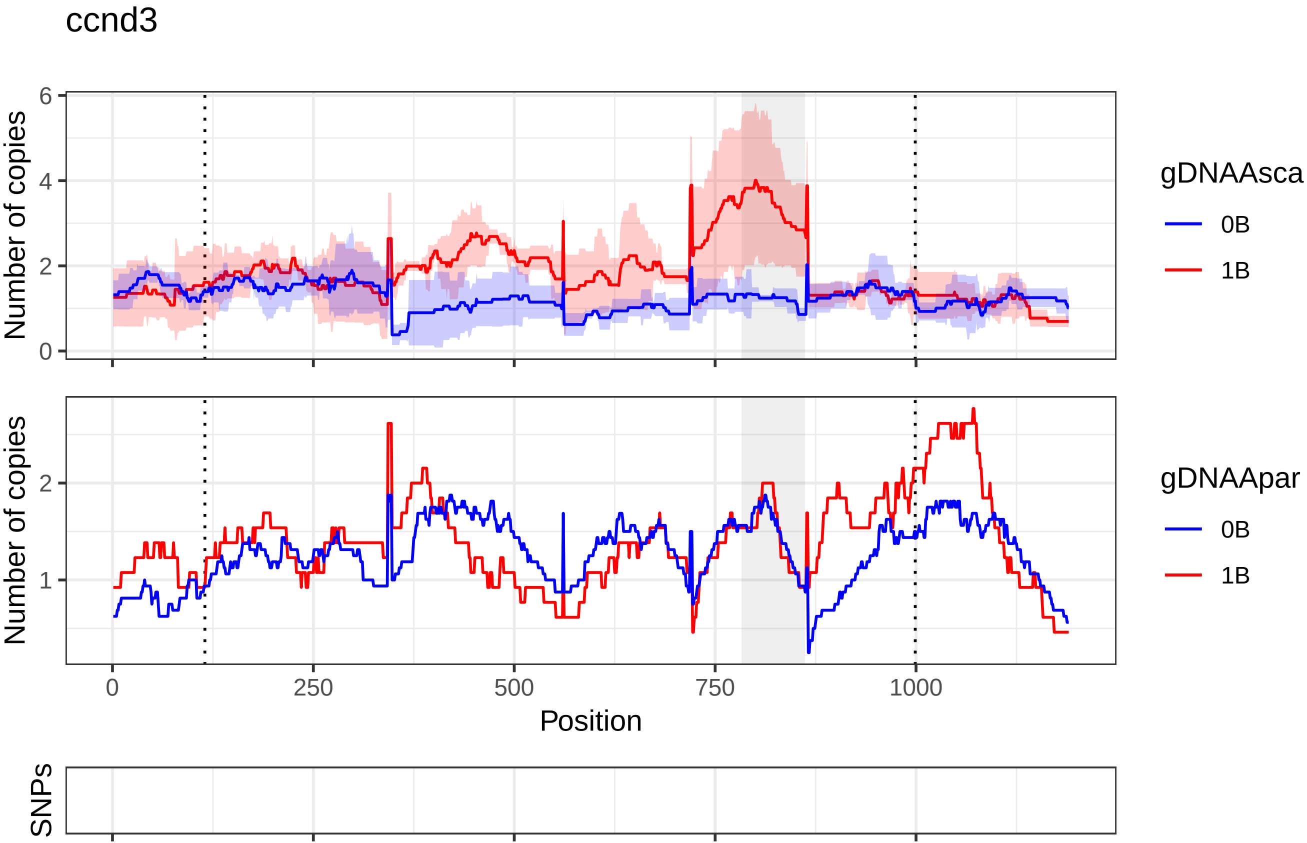
ccnd3
Number of copies
6
4
2
0
gDNAAsca
0B
1B
Number of copies
2
1
gDNAApar
0B
1B
0
250
500
750
1000
Position
SNPs

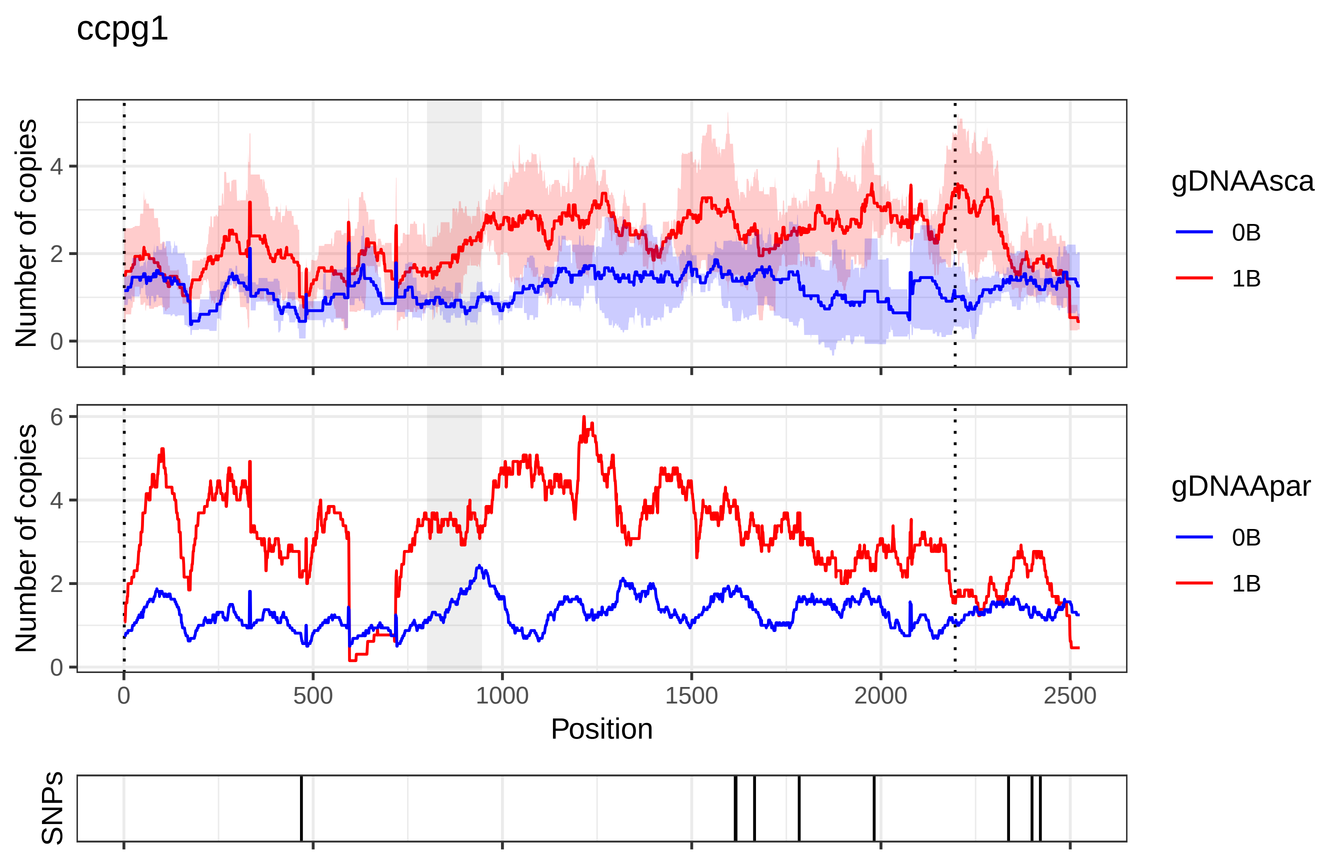

ccpg1
Number of copies
gDNAAsca
0B
1B
gDNAApar
0B
1B
Position
0
500
1000
1500
2000
2500
0
2
4
6
SNPs

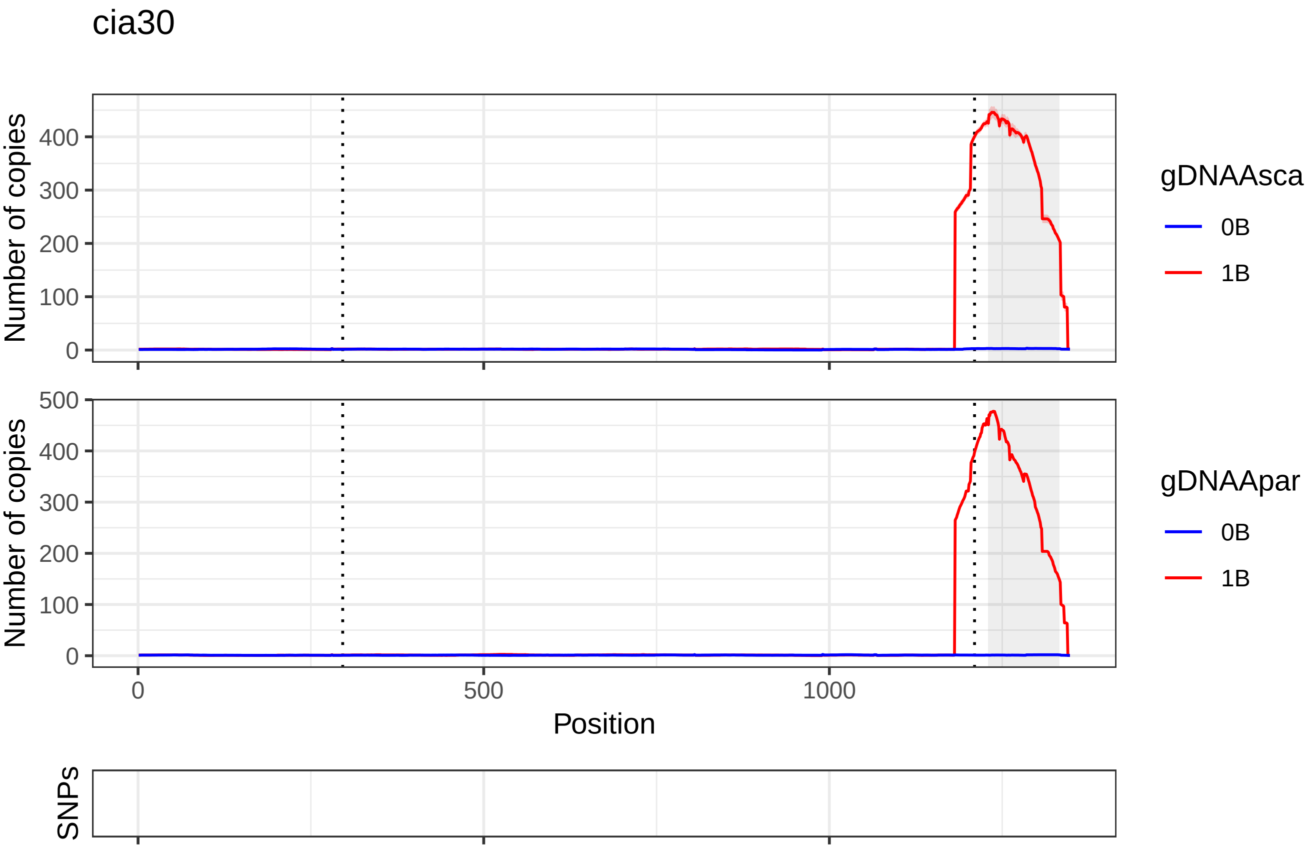

cia30
Number of copies
400
300
200
100
0
gDNAAsca
0B
1B
Number of copies
500
400
300
200
100
0
gDNAApar
0B
1B
0
500
1000
Position
SNPs

g2e3

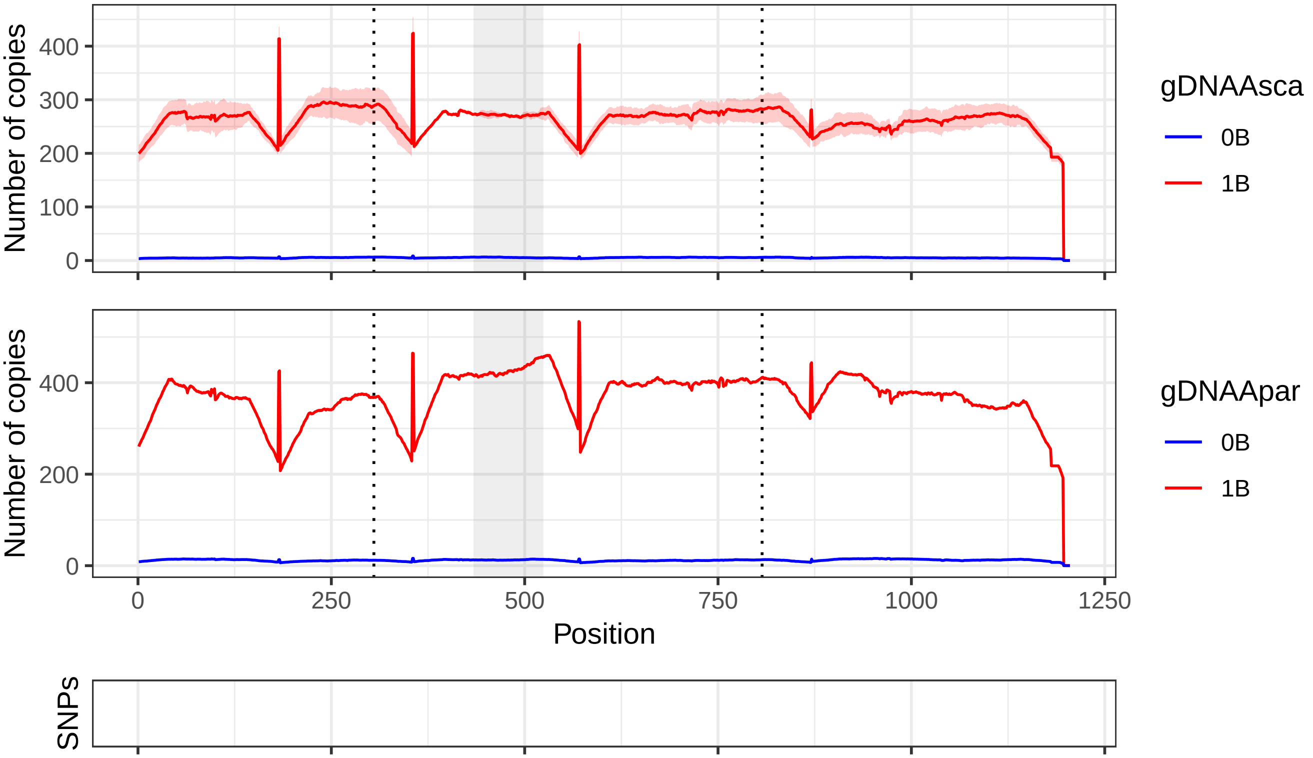

Number of copies
400
300
200
100
0
gDNAAsca
0B
1B
Number of copies
400
200
0
gDNAApar
0B
1B
0
250
500
750
1000
1250
Position
SNPs

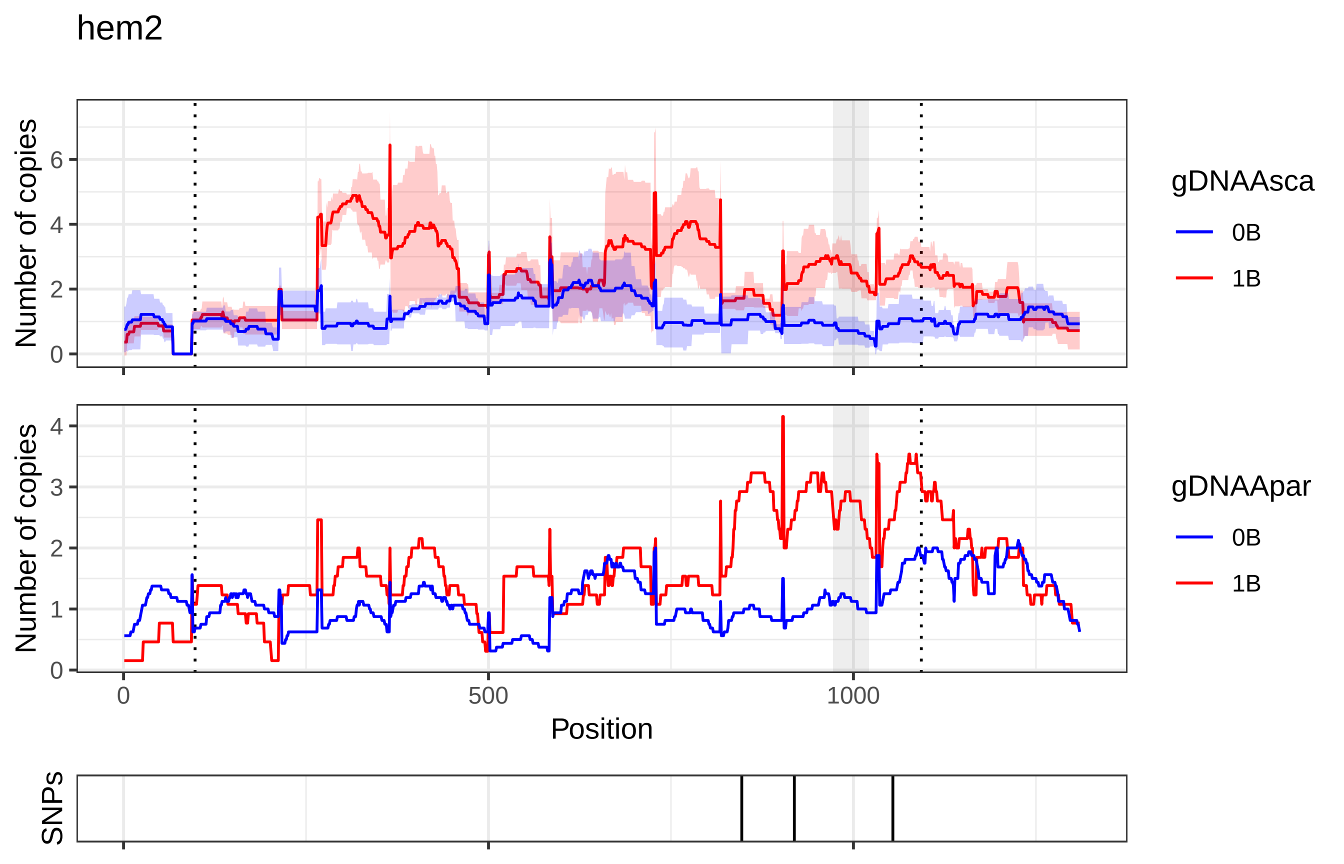

hem2
Number of copies
0
2
4
6
gDNAAsca
0B
1B
Number of copies
0
1
2
3
4
gDNAApar
0B
1B
0
500
1000
Position
SNPs

lap4a

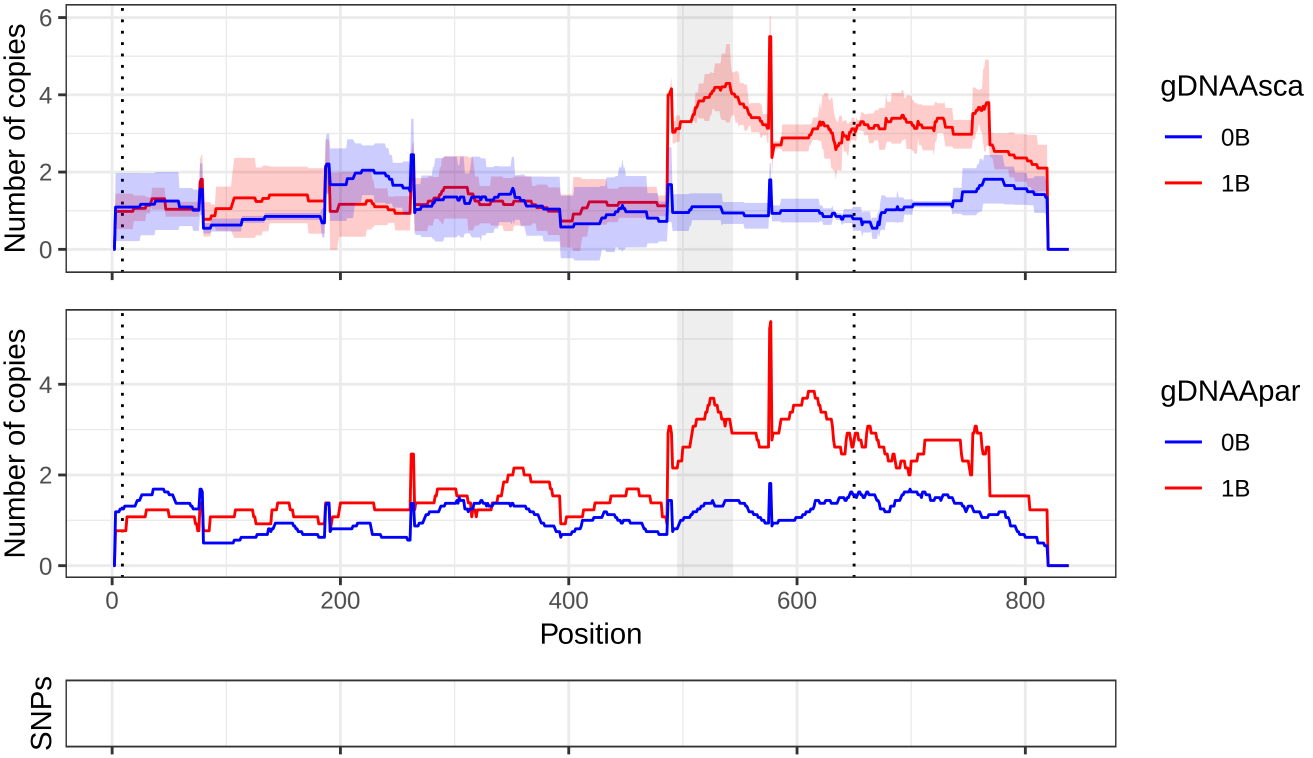

Number of copies
6
4
2
0
gDNAAsca
0B
1B
Number of copies
4
2
0
gDNAApar
0B
1B
0
200
400
600
800
Position
SNPs

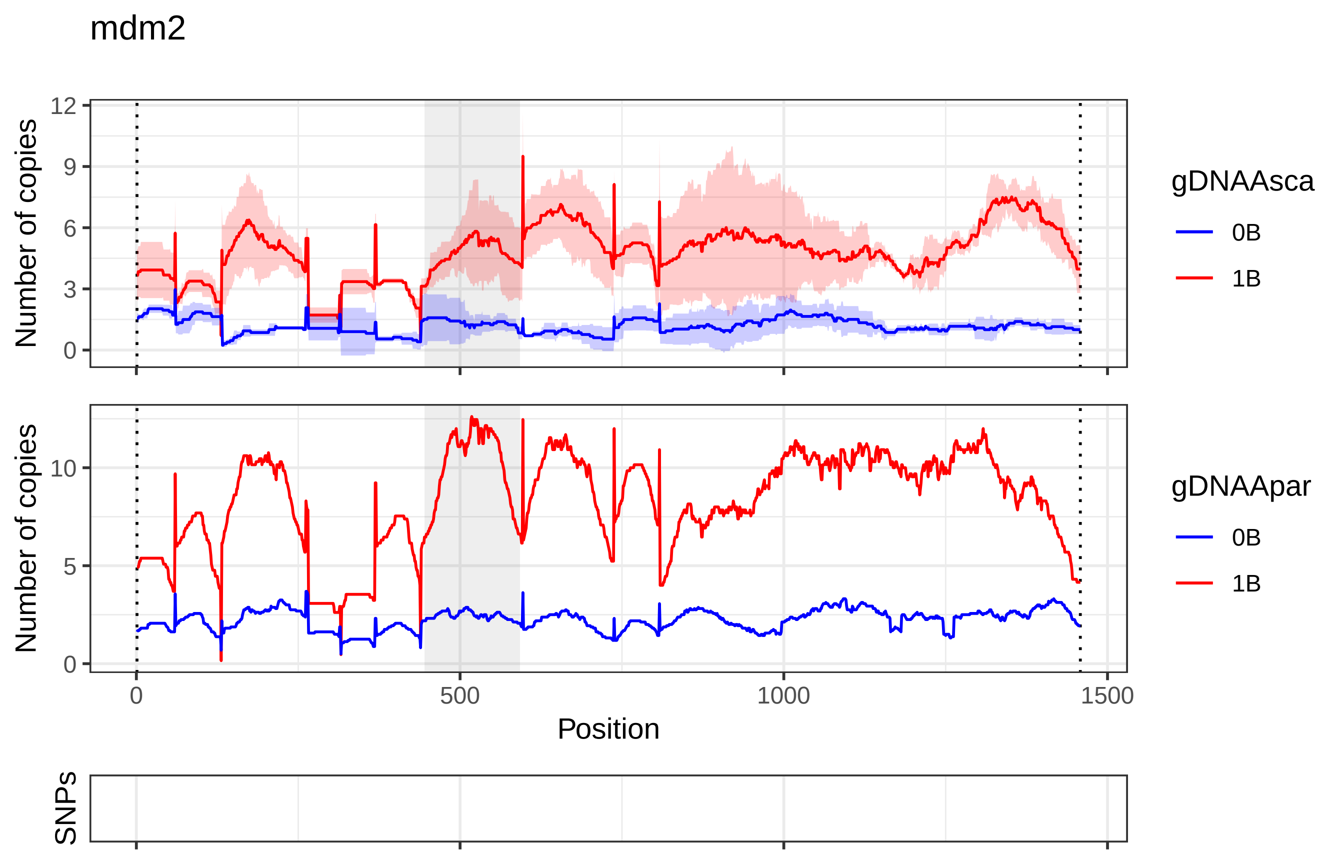

mdm2
Number of copies
gDNAAsca
0B
1B
gDNAApar
Position
SNPs
0
3
6
9
12
5
10
500
1000
1500

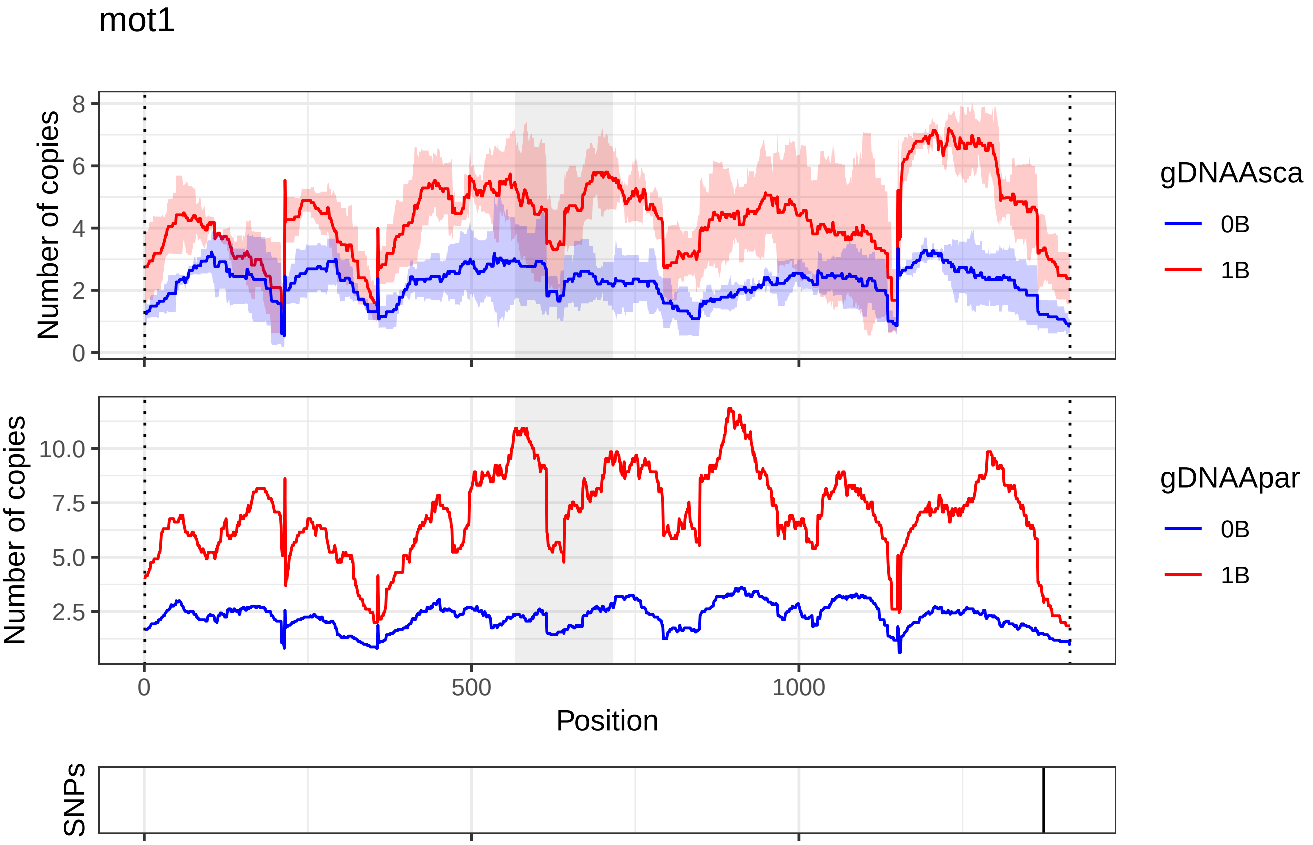

mot1
Number of copies
8
6
4
2
0
gDNAAsca
0B
1B
Number of copies
10.0
7.5
5.0
2.5
gDNAApar
0B
1B
0
500
1000
Position
SNPs

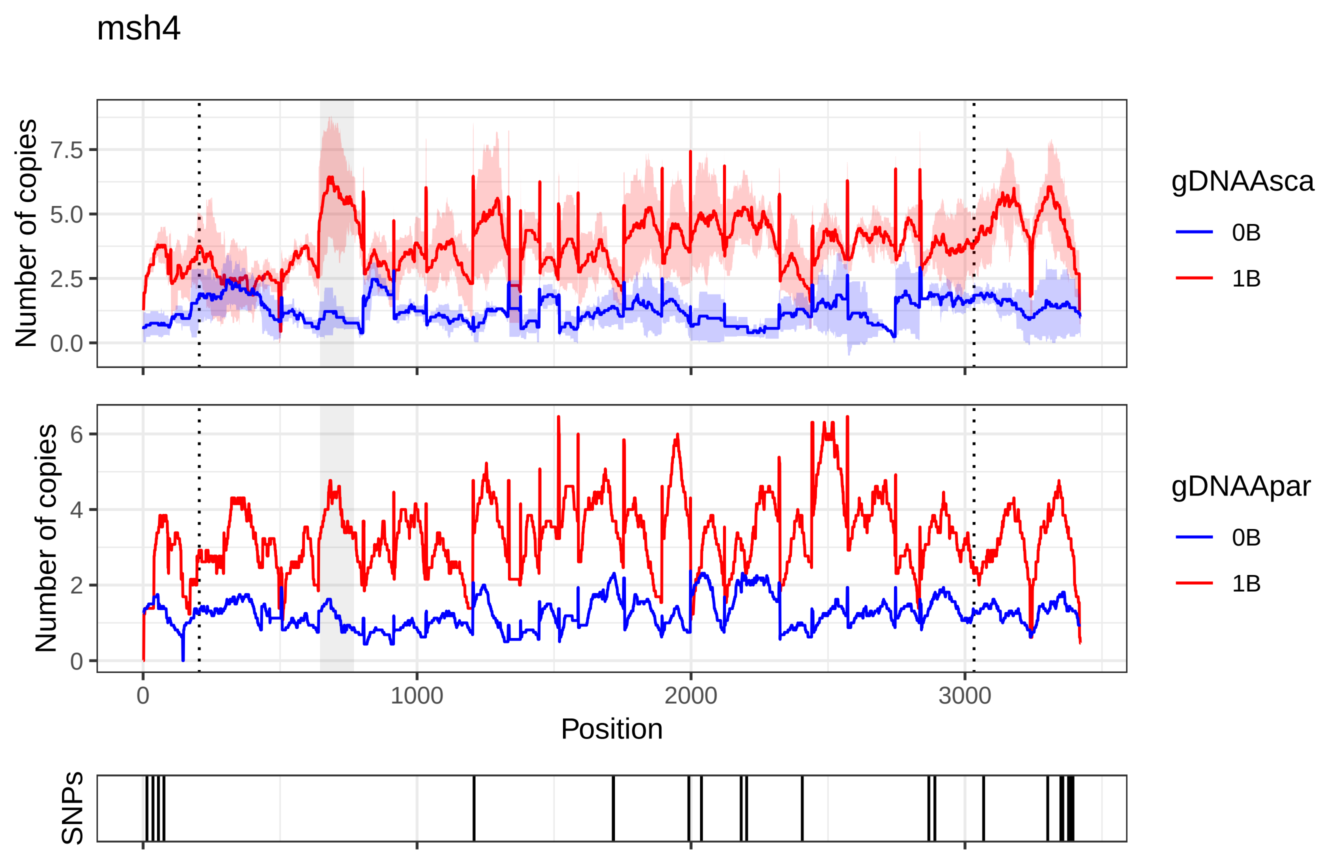
msh4
Number of copies
7.5
5.0
2.5
0.0
gDNAAsca
0B
1B
Number of copies
6
4
2
0
gDNAApar
0B
1B
0
1000
2000
3000
Position
SNPs

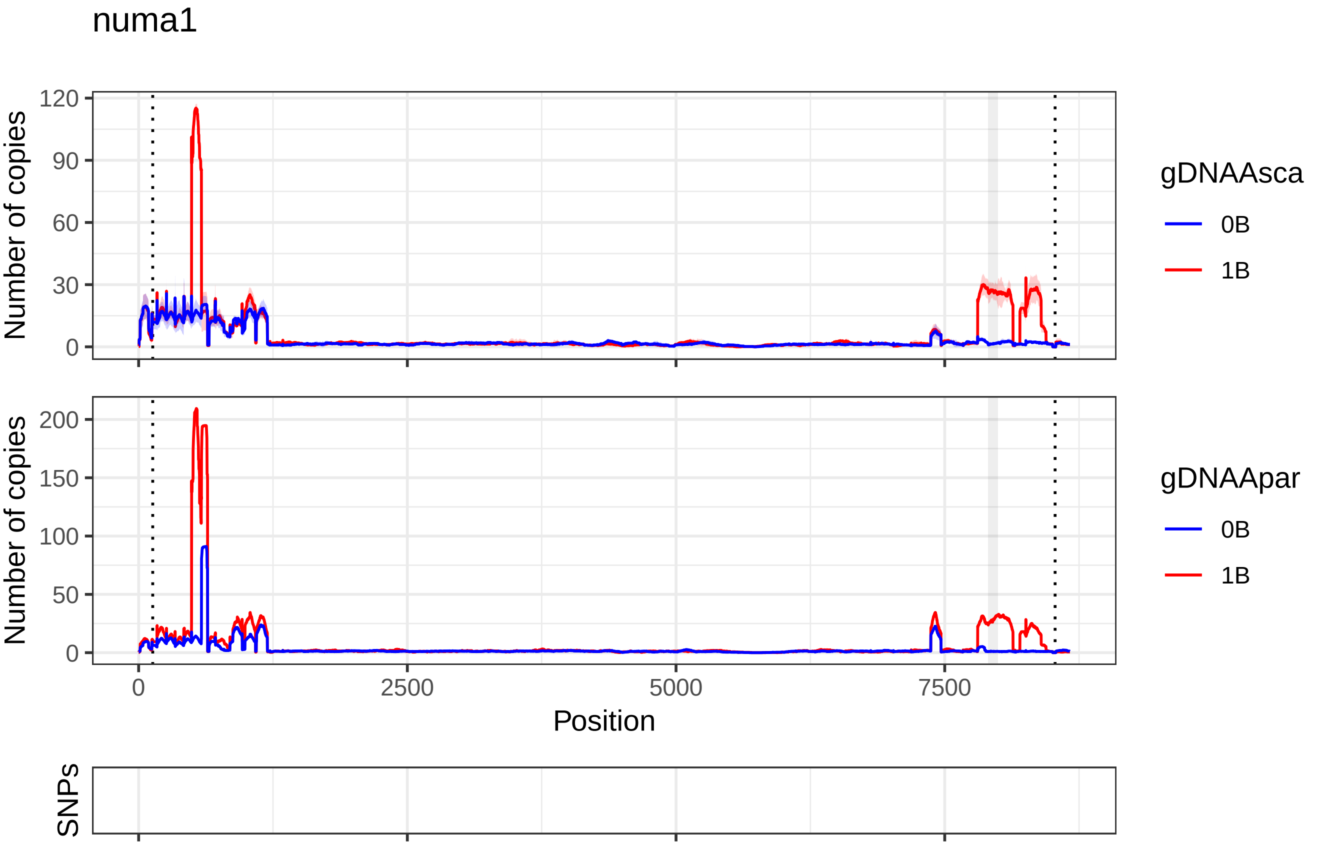
numa1
Number of copies
120
90
60
30
0
gDNAAsca
0B
1B
Number of copies
200
150
100
50
0
gDNAApar
0B
1B
0
2500
5000
7500
Position
SNPs

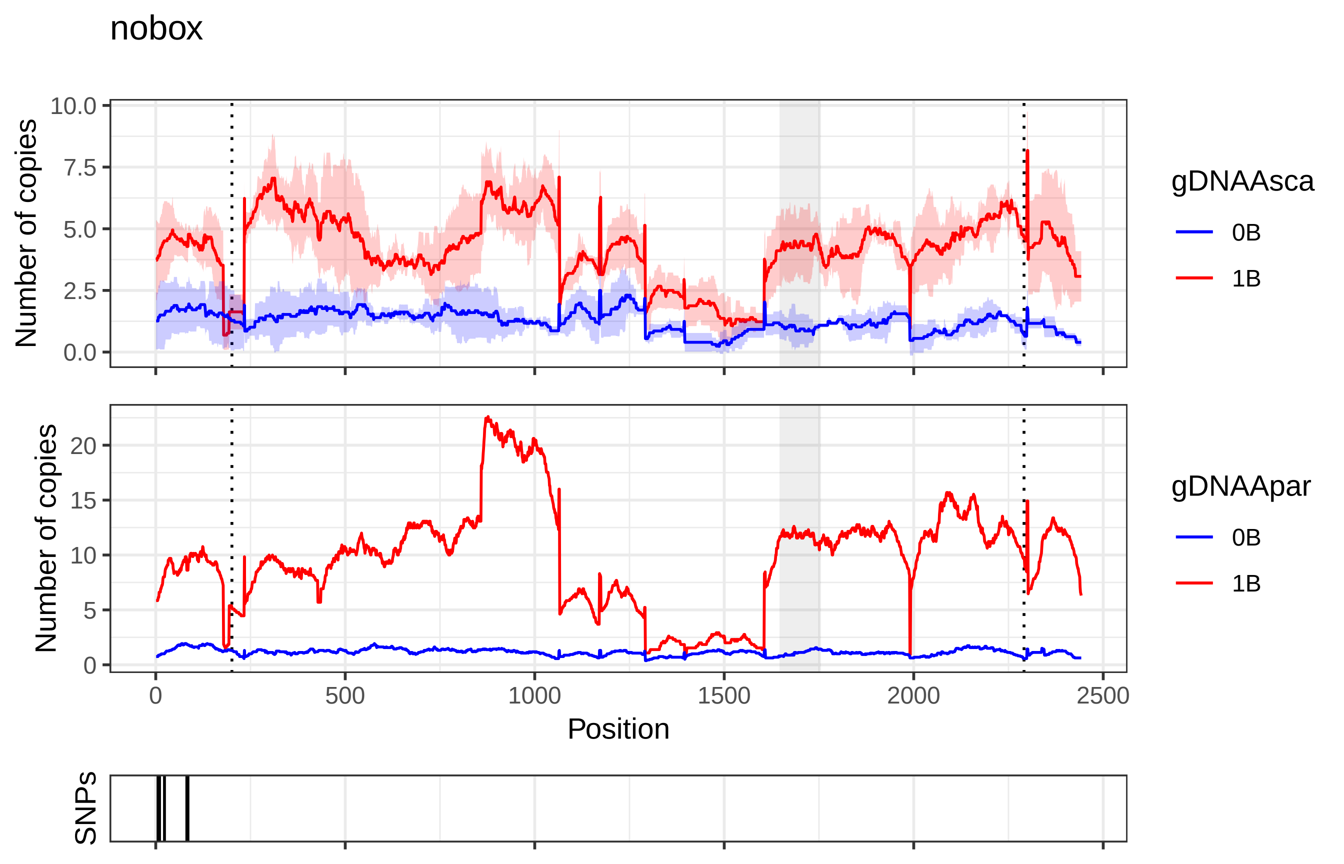

nobox
10.0
7.5
5.0
2.5
0.0
Number of copies
gDNAAsca
0B
1B
20
15
10
5
0
Number of copies
gDNAApar
0B
1B
0
500
1000
1500
2000
2500
Position
SNPs

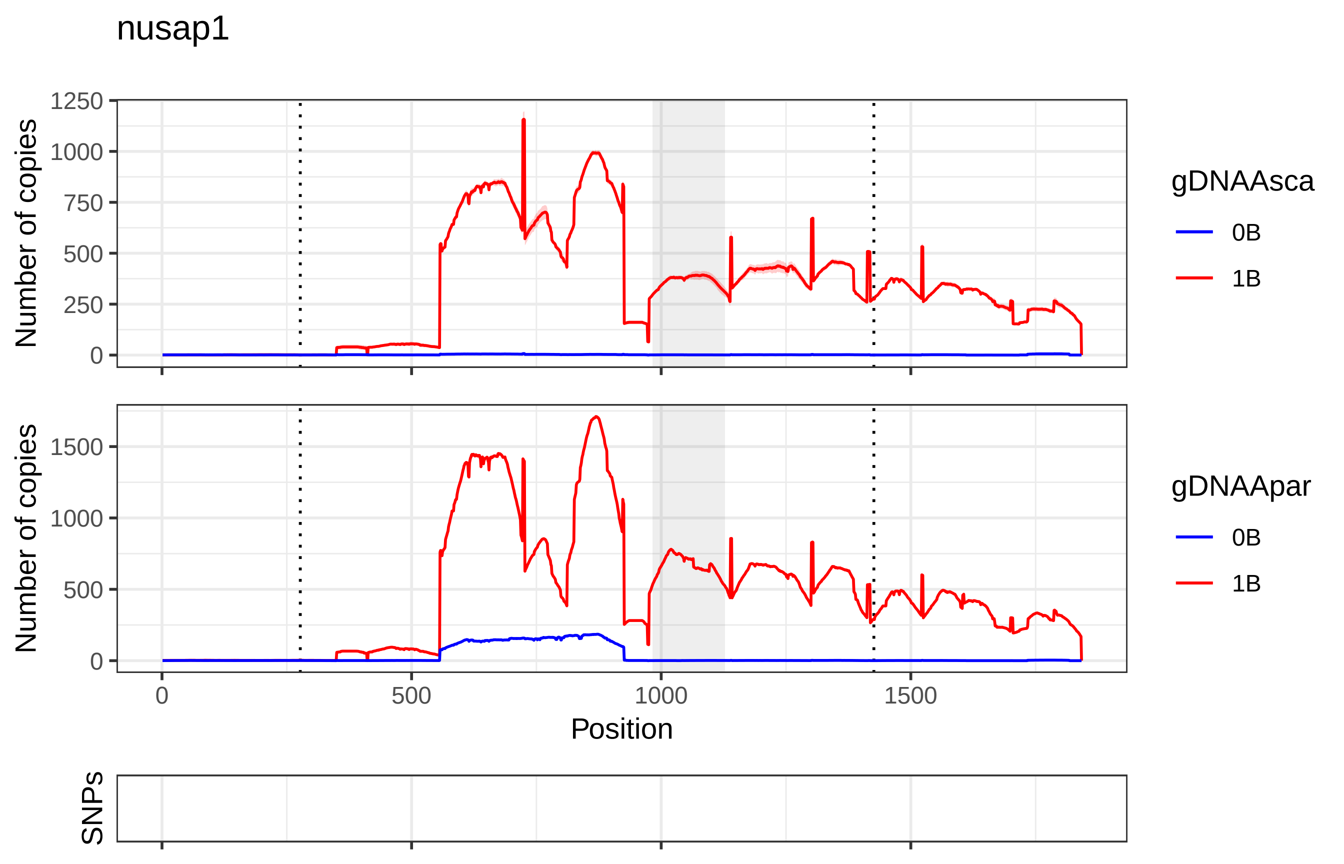
nusap1
Number of copies
1250
1000
750
500
250
0
gDNAAsca
0B
1B
Number of copies
1500
1000
500
0
gDNAApar
0B
1B
0
500
1000
1500
Position
SNPs

rnf17

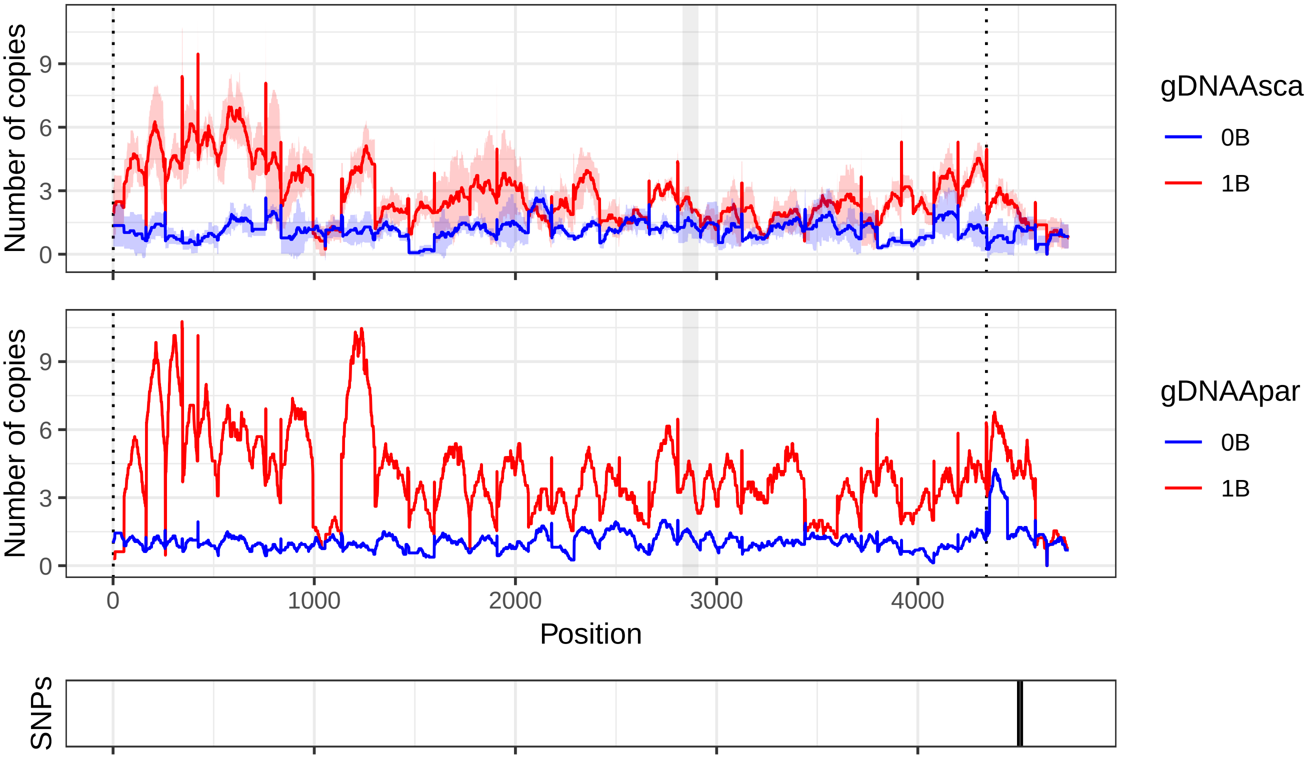

Number of copies
9
6
3
0
gDNAAsca
0B
1B
Number of copies
9
6
3
0
gDNAApar
0B
1B
0
1000
2000
3000
4000
Position
SNPs

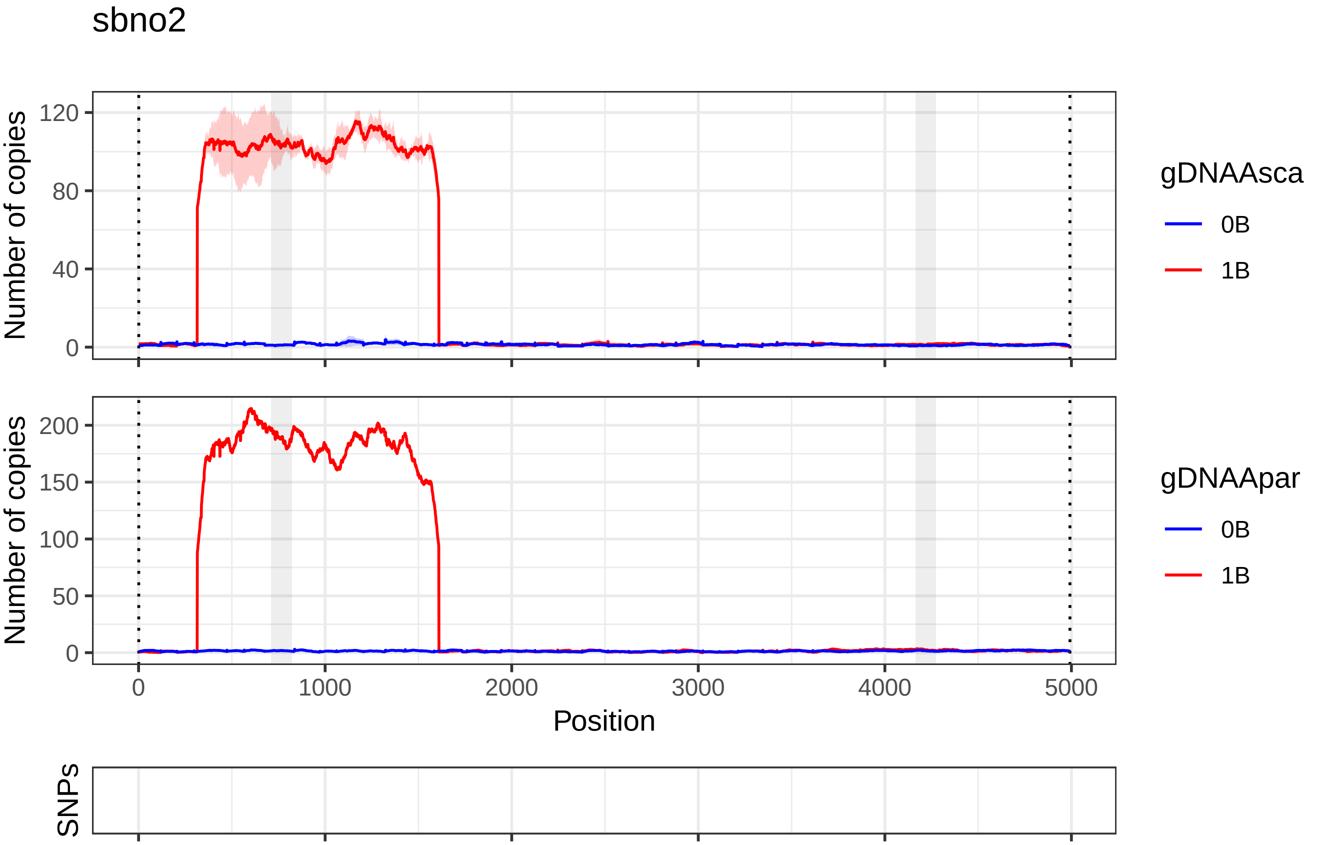
sbno2
Number of copies
120
80
40
0
gDNAAsca
0B
1B
Number of copies
200
150
100
50
0
gDNAApar
0B
1B
0
1000
2000
3000
4000
5000
Position
SNPs

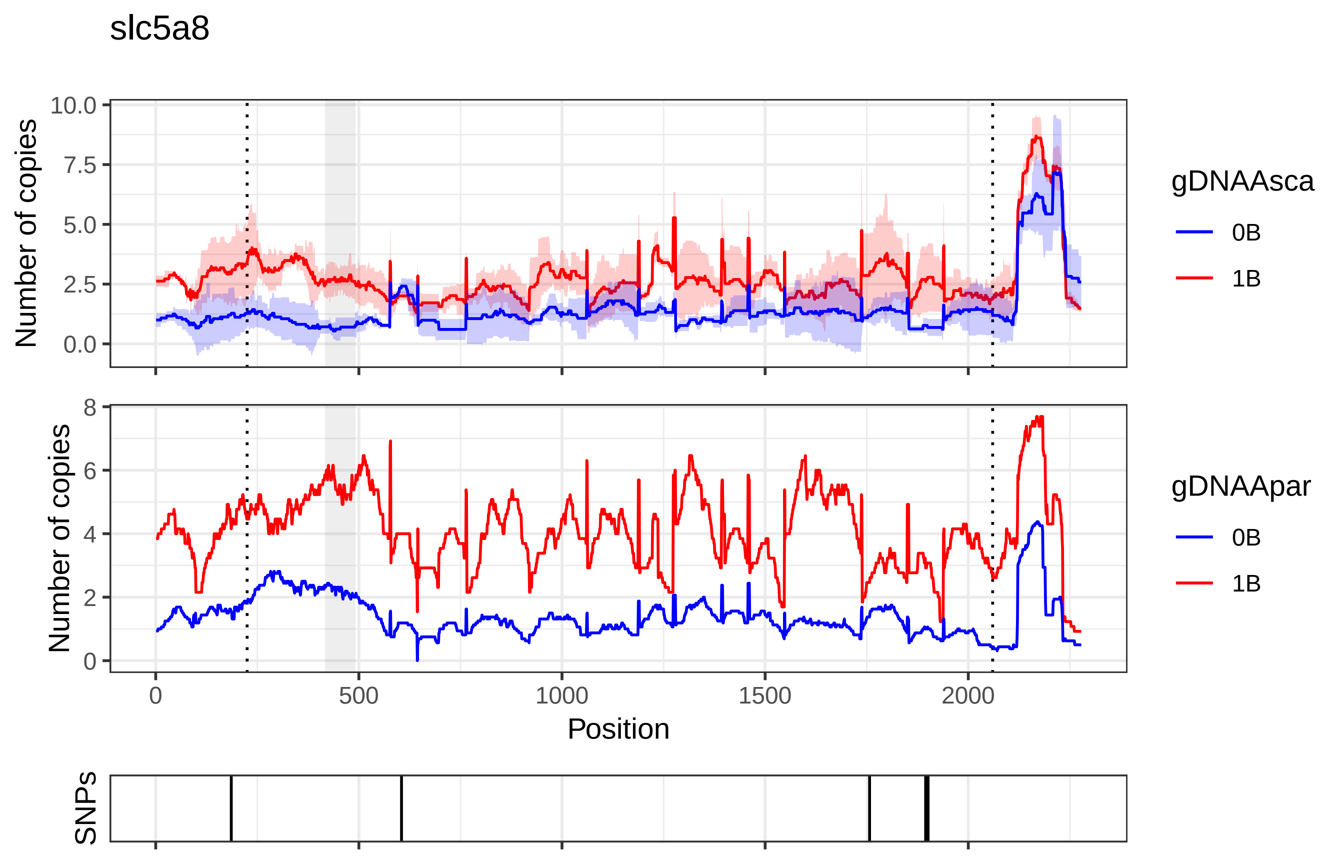

slc5a8
Number of copies
10.0
7.5
5.0
2.5
0.0
gDNAAsca
0B
1B
Number of copies
8
6
4
2
0
gDNAApar
0B
1B
0
500
1000
1500
2000
Position
SNPs

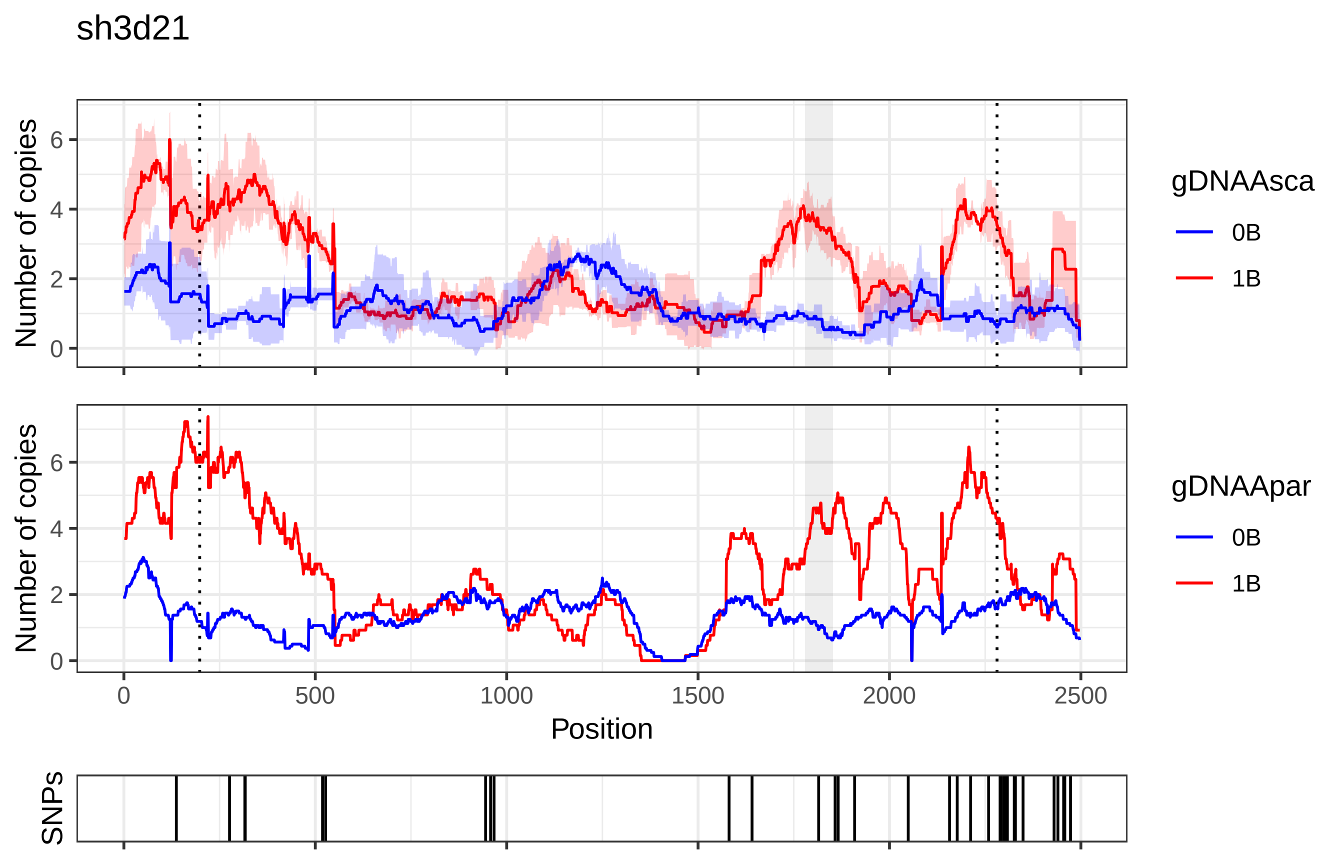
sh3d21
Number of copies
gDNAAsca
0B
1B
Number of copies
gDNAApar
0B
1B
0
500
1000
1500
2000
2500
Position
SNPs

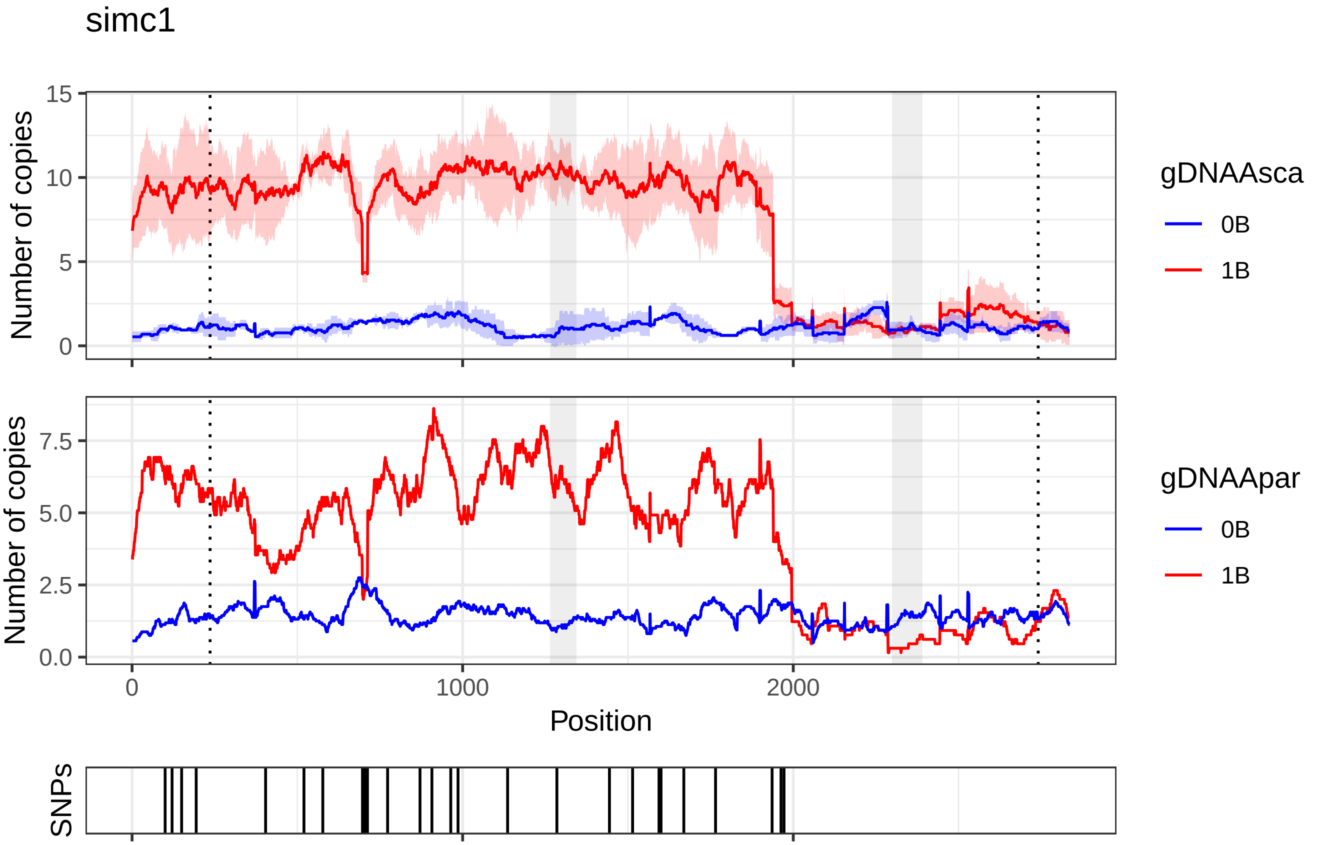
simc1
Number of copies
15
10
5
0
gDNAAsca
0B
1B
Number of copies
7.5
5.0
2.5
0.0
gDNAApar
0B
1B
0
1000
2000
Position
SNPs

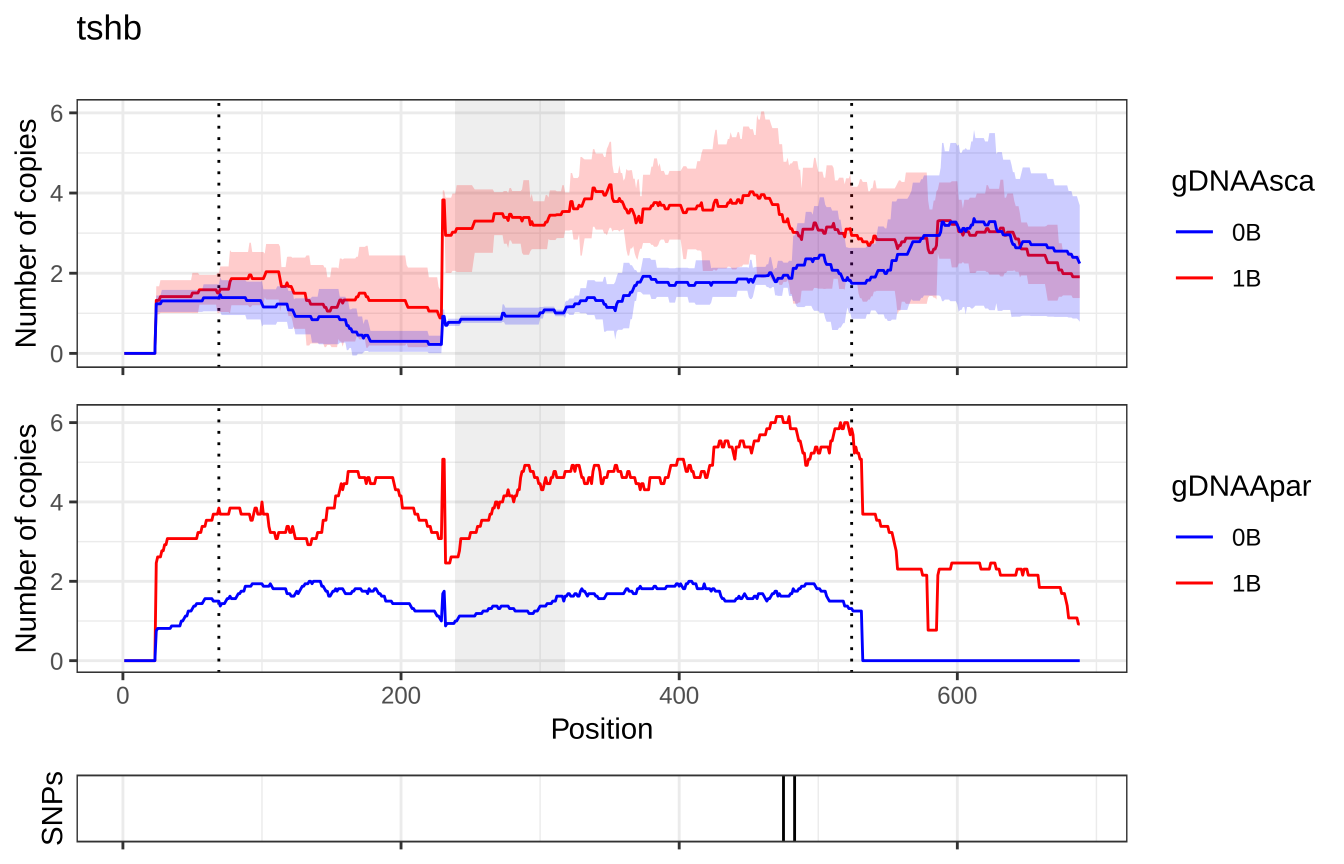
tshb
Number of copies
6
4
2
0
gDNAAsca
0B
1B
gDNAApar
0B
1B
0
200
400
600
Position
SNPs

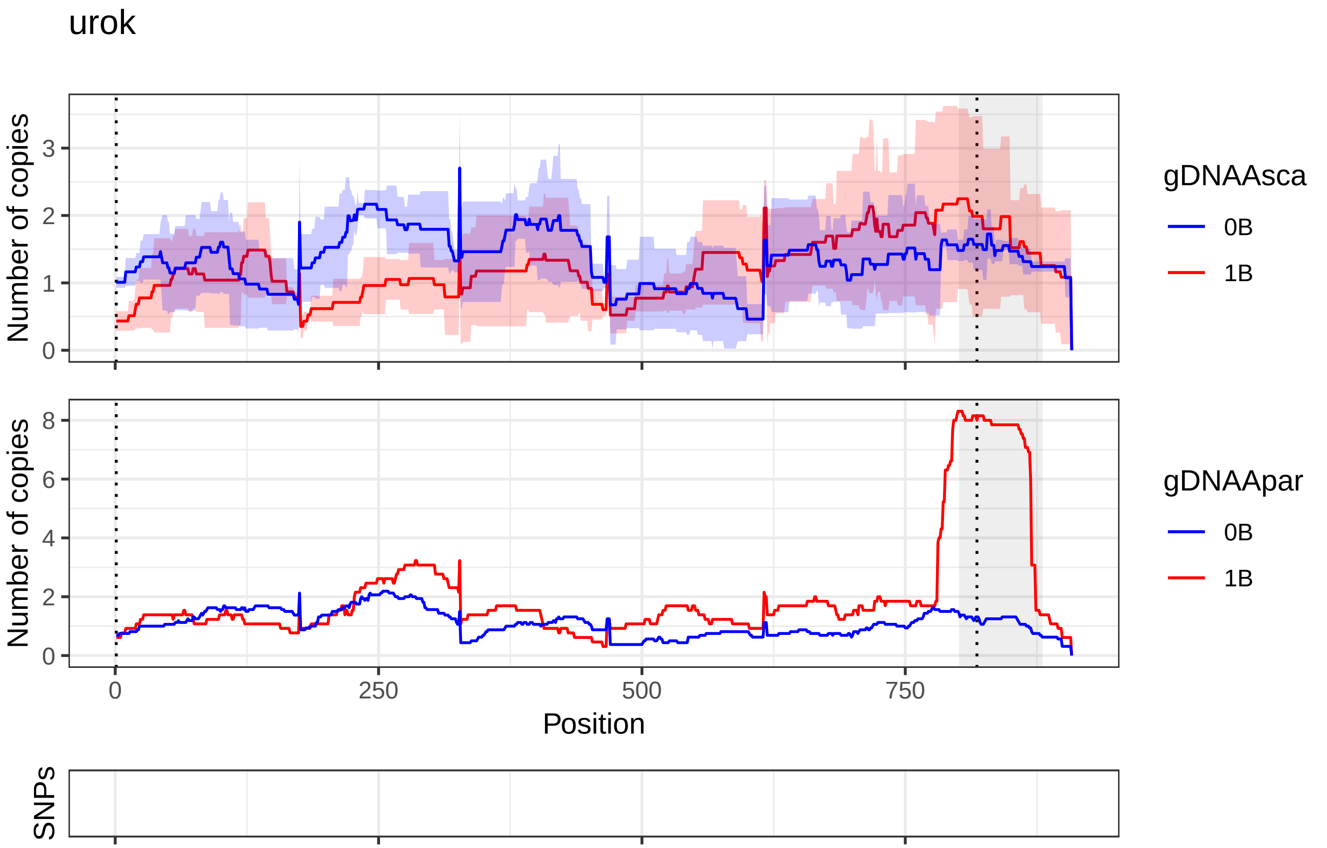

urok
Number of copies
gDNAAsca
0B
1B
gDNAApar
0B
1B
0
250
500
750
Position
SNPs

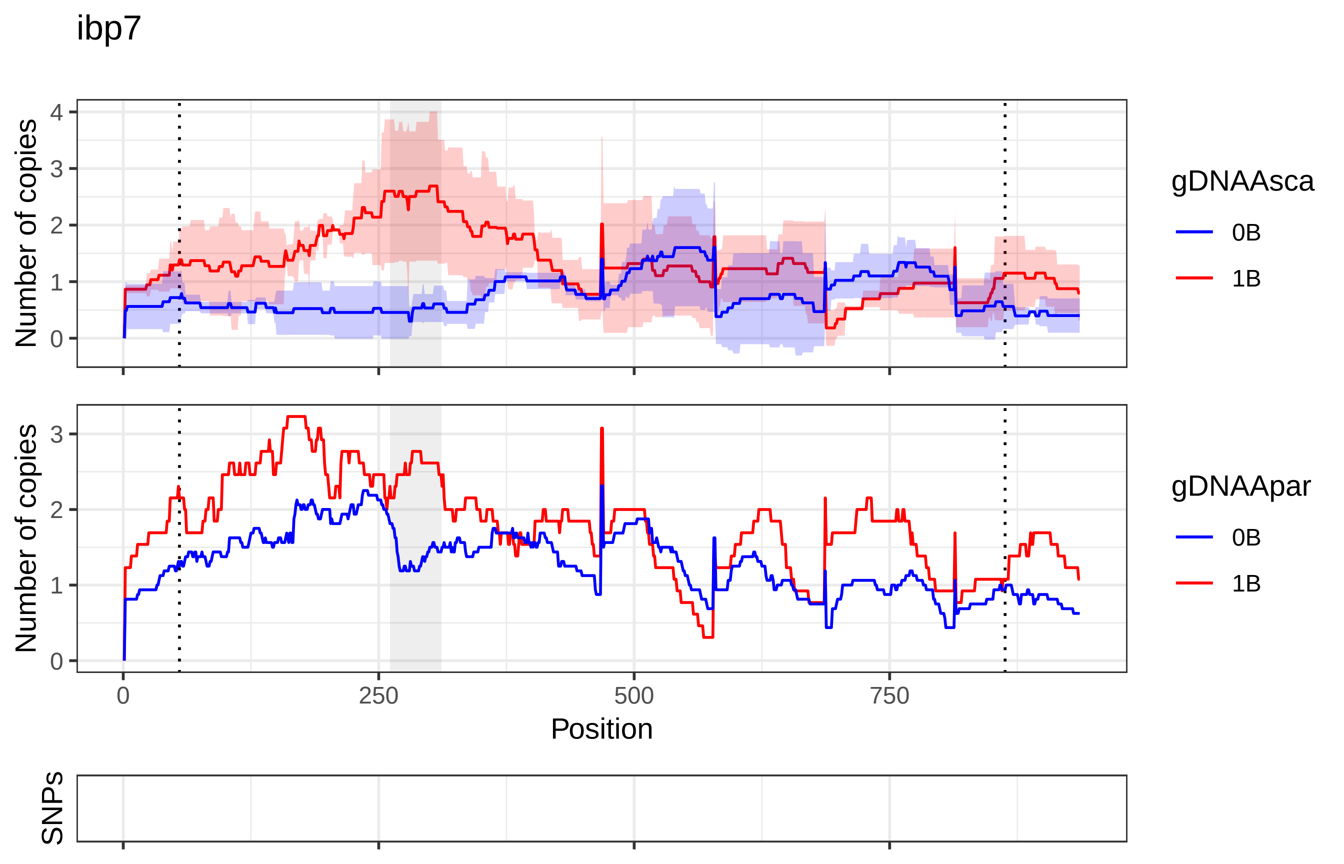
ibp7
Number of copies
4
3
2
1
0
gDNAAsca
0B
1B
Number of copies
3
2
1
0
gDNAApar
0B
1B
0
250
500
750
Position
SNPs

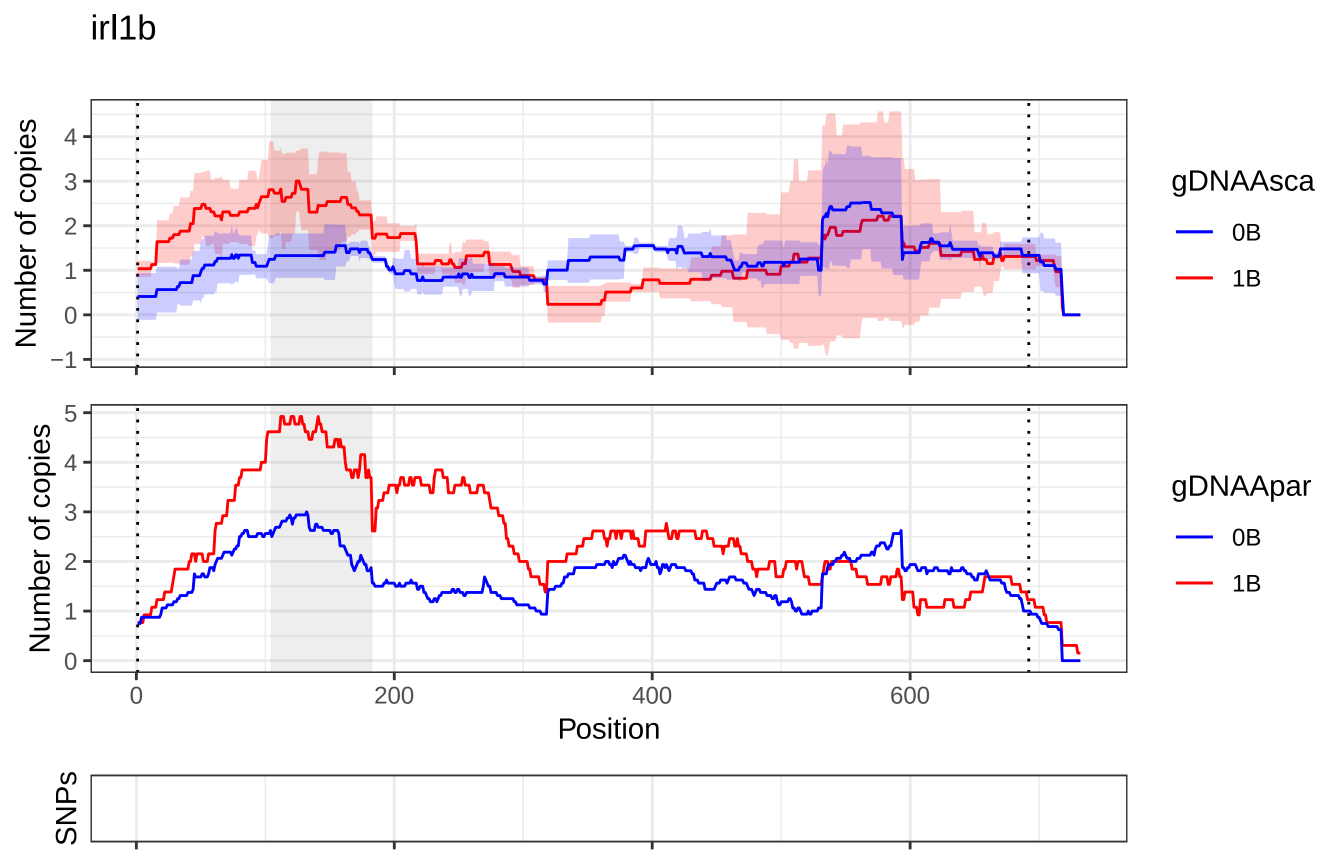
irl1b
Number of copies
gDNAAsca
0B
1B
gDNAApar
0B
1B
Position
SNPs
0
200
400
600

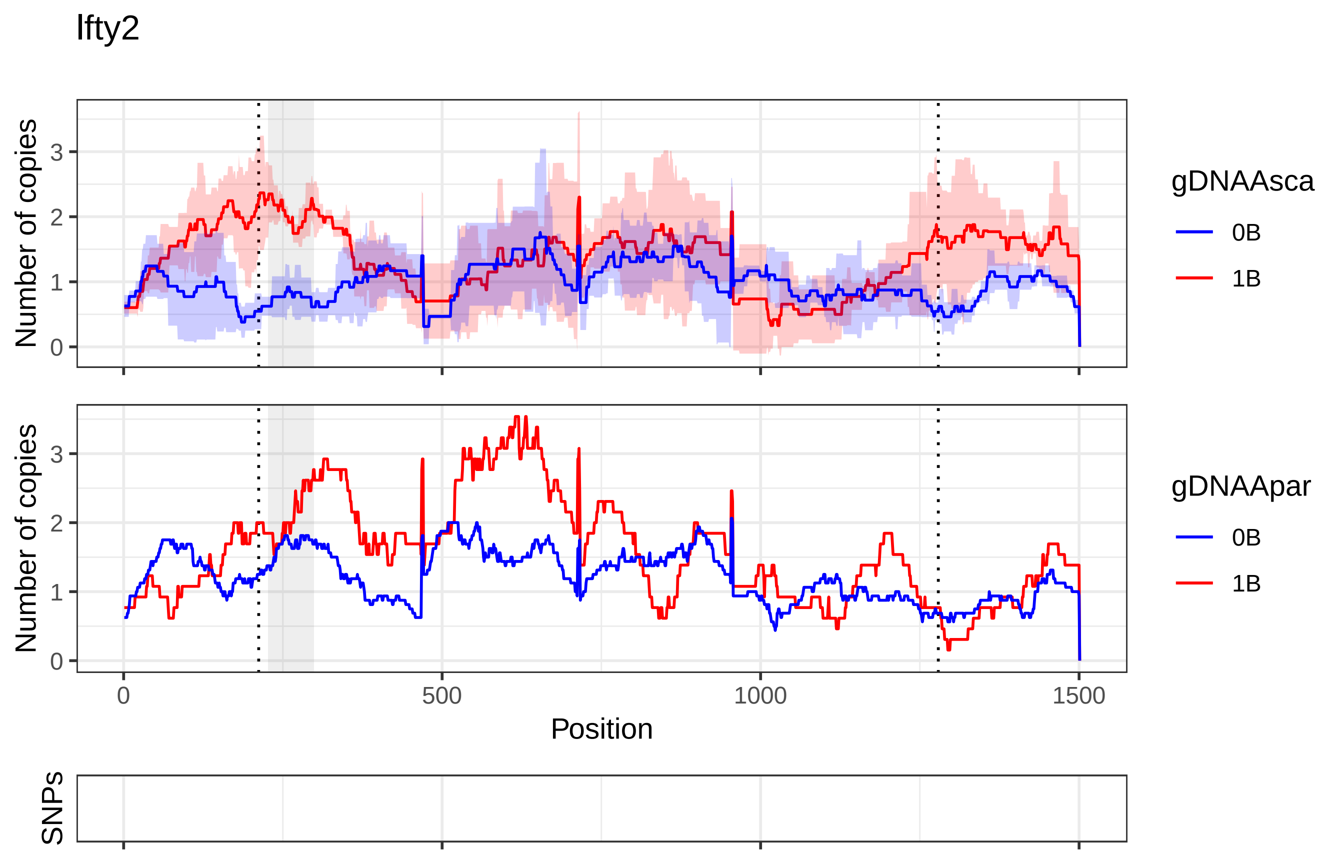

lfty2
Number of copies
0
1
2
3
gDNAAsca
0B
1B
gDNAApar
0B
1B
0
500
1000
1500
Position
SNPs

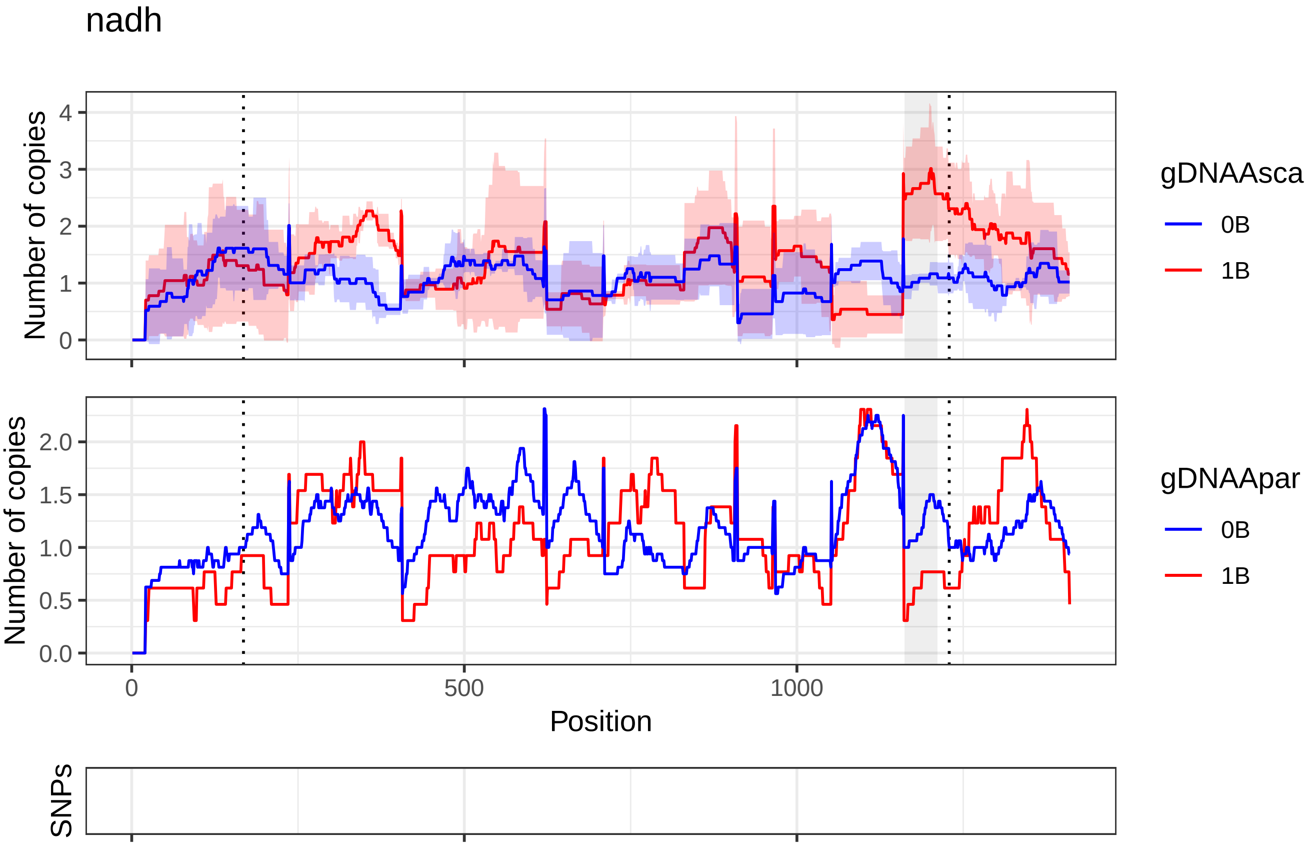
nadh
Number of copies
4
3
2
1
0
gDNAAsca
0B
1B
Number of copies
2.0
1.5
1.0
0.5
0.0
gDNAApar
0B
1B
0
500
1000
Position
SNPs

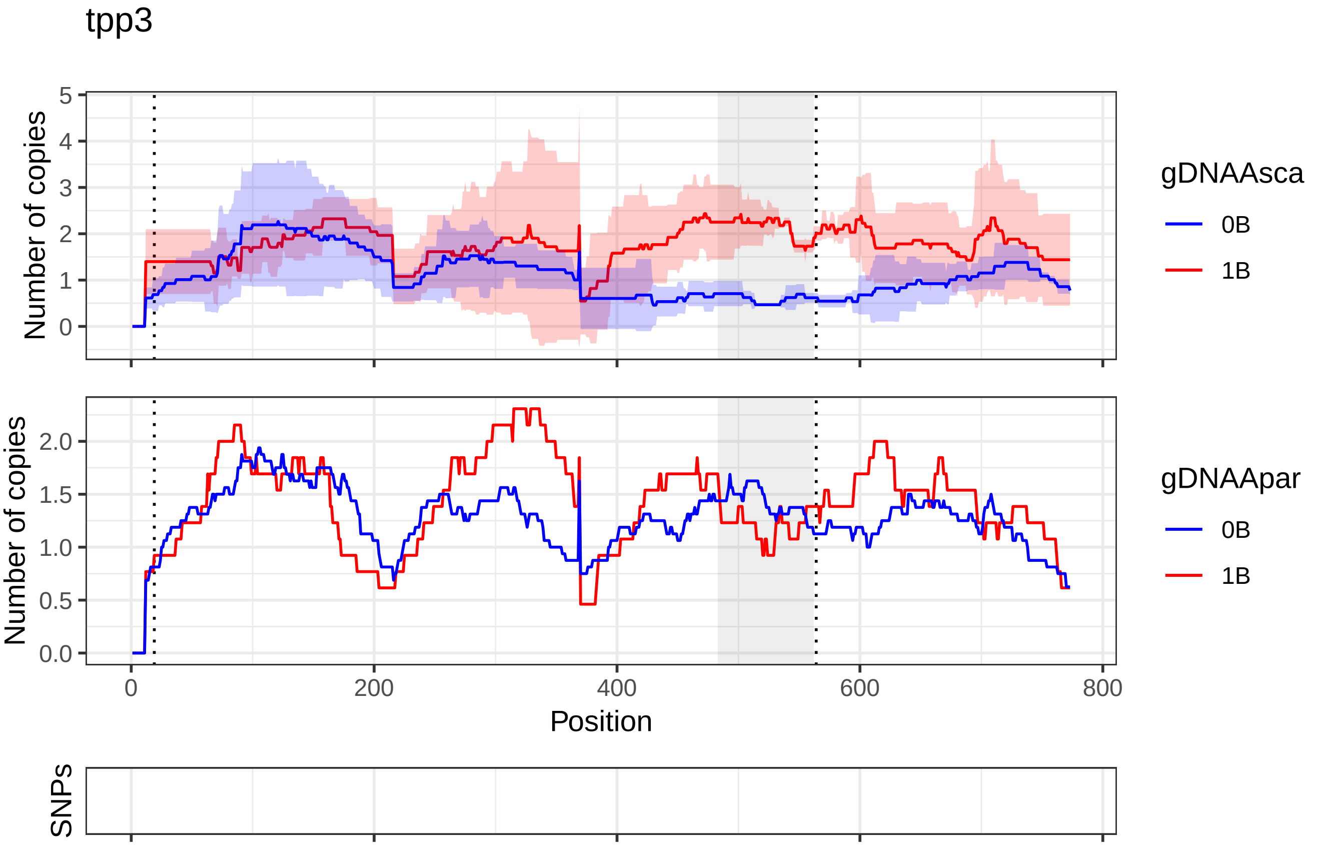

tpp3
Number of copies
5
4
3
2
1
0
gDNAAsca
0B
1B
Number of copies
2.0
1.5
1.0
0.5
0.0
gDNAApar
0B
1B
0
200
400
600
800
Position
SNPs

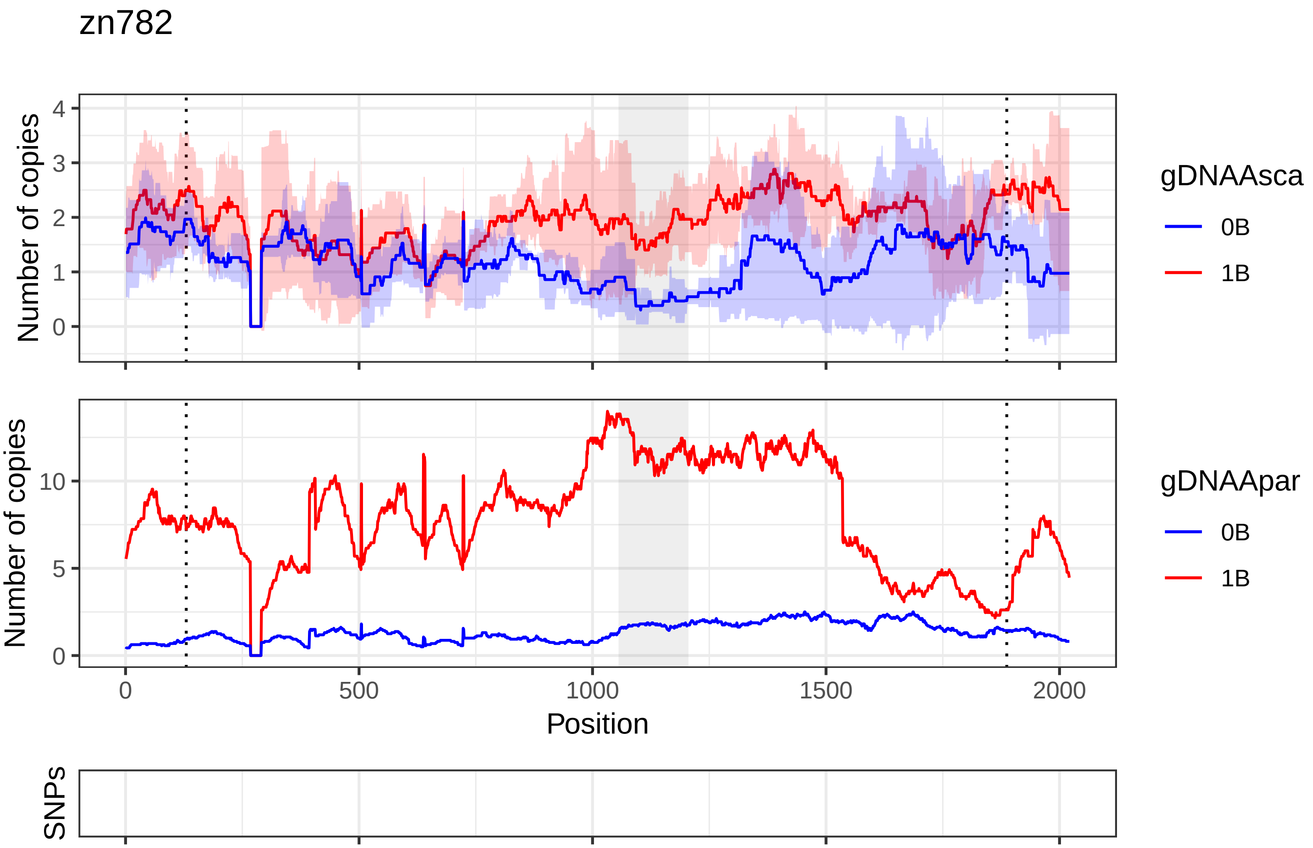
zn782
Number of copies
gDNAAsca
0B
1B
gDNAApar
0B
1B
0
500
1000
1500
2000
Position
SNPs
